# DNA replication stress and mitotic catastrophe mediate sotorasib addiction in KRAS^G12C^-mutant cancer

**DOI:** 10.1186/s12929-023-00940-4

**Published:** 2023-06-29

**Authors:** Li-Wen Chiou, Chien-Hui Chan, Yu-Ling Jhuang, Ching-Yao Yang, Yung-Ming Jeng

**Affiliations:** 1 Graduate Institute of Pathology, College of Medicine, National Taiwan University, Taipei, Taiwan; 2 Department of Surgery, National Taiwan University Hospital, 7 Chung-Shan South Road, Taipei, 100 Taiwan; 3 Department of Surgery, College of Medicine, National Taiwan University, Taipei, Taiwan; 4 Department of Pathology, National Taiwan University Hospital, 7 Chung-Shan South Road, Taipei, 100 Taiwan

**Keywords:** Sotorasib, Drug addiction, KRAS, Replication stress, Mitotic catastrophe

## Abstract

**Background:**

Sotorasib is the first KRAS^G12C^ inhibitor approved by the US Food and Drug Administration for treating KRAS^G12C^-mutant non-small-cell lung cancer (NSCLC). Clinical trials on the therapeutic use of sotorasib for cancer have reported promising results. However, KRAS^G12C^-mutant cancers can acquire resistance to sotorasib after treatment. We incidentally discovered that sotorasib-resistant (SR) cancer cells are addicted to this inhibitor. In this study, we investigated the mechanisms underlying sotorasib addiction.

**Methods:**

Sotorasib-resistant cells were established using KRAS^G12C^-mutant pancreatic cancer and NSCLC cell lines. Cell viability in the presence or absence of sotorasib and in combination with multiple inhibitors was assessed through proliferation assay and annexin V/propidium iodide (PI) flow cytometry assays. The mechanisms underlying drug addiction were elucidated through 5-bromo-2′-deoxyuridine (BrdU) incorporation assay, immunofluorescence staining, time-lapse microscopy, and comet assay. Furthermore, a subcutaneous xenograft model was used to demonstrate sotorasib addiction in vivo.

**Results:**

In the absence of sotorasib, the sotorasib-resistant cells underwent p21^Waf1^/^Cip1^-mediated cell cycle arrest and caspase-dependent apoptosis. Sotorasib withdrawal resulted in robust activation of mitogen-activated protein kinase (MAPK) pathway, inducing severe DNA damage and replication stress, which activated the DNA damage response (DDR) pathway. Persistent MAPK pathway hyperactivation with DDR exhaustion led to premature mitotic entry and aberrant mitosis, followed by micronucleus and nucleoplasmic bridge formation. Pharmacologic activation of the MAPK pathway with a type I BRAF inhibitor could further enhance the effects of sotorasib withdrawal on sotorasib-resistant cancer cells both in vitro and in vivo.

**Conclusions:**

We elucidated the mechanisms underlying the sotorasib addiction of cancer cells. Sotorasib addiction appears to be mediated through MAPK pathway hyperactivity, DNA damage, replication stress, and mitotic catastrophe. Moreover, we devised a therapeutic strategy involving a type I BRAF inhibitor to strengthen the effects of sotorasib addiction; this strategy may provide clinical benefit for patients with cancer.

**Supplementary Information:**

The online version contains supplementary material available at 10.1186/s12929-023-00940-4.

## Background

*Kirsten rat sarcoma viral oncogene homologue* (*KRAS*)—one of the most prevalent cancer driver genes—is mutated in approximately 95% of pancreatic cancer [1], 50% of colorectal cancer [2], and 20% of non-small-cell lung cancer (NSCLC) [3] cases. The activity of KRAS is regulated through its cycling between an inactive GDP-bound and an active GTP-bound state [4, 5]. Mutations at codons 12, 13, or 61 prevent GTPase-activating protein–induced GTP hydrolysis and trap KRAS in the active GTP-bound state; this lead to the constitutive activation of downstream signal pathways, such as the mitogen-activated protein kinase (MAPK) and phosphatidylinositol 3-kinase (PI3K) pathways [6].

Because of the high mutation rate of *KRAS* in cancer, many studies have explored treatments targeting KRAS or its downstream pathways. However, until recently, none of these treatments are clinical applicable. Thus, KRAS has long been considered “undruggable” [7]. Recently, several KRAS^G12C^-targeting drugs, such as ARS-1620, sotorasib (AMG510), adagrasib (MRTX849), JNJ-74699157, and LY3499446, have been developed [8]. By accessing the switch-II pocket and covalently binding to GDP-bound KRAS^G12C^ without affecting wild-type KRAS, these drugs arrest KRAS^G12C^ in its inactive state, resulting in the inhibition of its downstream pathways [9]. In a phase II clinical trial (NCT03600883), 37.1% of NSCLC patients who received sotorasib exhibited an objective response to the treatment; the disease was controlled in 80.6% of the patients [10]. Therefore, in 2021, the therapeutic use of sotorasib was approved by the US Food and Drug Administration for KRAS^G12C^-mutant locally advanced or metastatic NSCLC [11].

Despite eliciting considerable initial treatment response, molecular targeted therapy typically cannot eradicate all tumor cells. Residual tumors eventually acquire drug resistance through genetic and nongenetic alternations, leading to cancer relapse. Zhao et al. observed genetic alterations in the post-treatment specimens of 27 (62%) of 43 patients who received sotorasib; these alterations were noted in *KRAS*, *neuroblastoma RAS viral oncogene homolog (NRAS)*, *v-Raf murine sarcoma viral oncogene homolog B (BRAF)*, *epidermal growth factor receptor (EGFR)*, *fibroblast growth factor receptor 2 (FGFR2)*, and *MYC* [12]. Nongenetic mechanisms underlying resistance to KRAS^G12C^ inhibitors include receptor tyrosine kinase activation, new KRAS protein synthesis, parallel pathway activation, and epithelial–mesenchymal transition [13–16]. Most mechanisms underlying drug resistance converge on reactivation of the MAPK pathway to bypass KRAS inhibition. Identifying the underlying drug resistance mechanisms is critical for improving treatment response and devising more effective combination strategies.

While studying the mechanisms underlying sotorasib resistance, we incidentally found reduced growth rate and increased cell death in KRAS^G12C^-mutant sotorasib-resistant cancer cells cultured in the absence of sotorasib. A similar “drug addiction” phenomenon was noted in BRAF-mutant cells treated with BRAF or mitogen-activated protein kinase kinase (MEK) inhibitors [17–20]. In this study, we investigated the mechanisms underlying sotorasib addiction, and found that robust MAPK activation after sotorasib withdrawal induced severe DNA damage and replication stress, resulting in the activation of DNA damage response (DDR) and p21^Waf1^/^Cip1^ (p21)-mediated cell cycle arrest. Moreover, persistent MAPK hyperactivation with DDR exhaustion eventually led to mitotic catastrophe. We further devised a novel therapeutic strategy for sotorasib-addicted cells by pharmacological enhancement of aberrant MAPK activation with a type I BRAF inhibitor, which effectively restrained cell growth both in vitro and in vivo*.*

## Methods

### Cell culture and reagents

The pancreatic cancer cell line MIA PaCa-2 was purchased from the Bioresource Collection and Research Center (Hsinchu, Taiwan). The lung cancer cell lines LU65 and NCI-H23 were obtained from the RIKEN Cell Bank (Ibaraki, Japan) and American Type Culture Collection (Manassas, VA, USA), respectively. MIA PaCa-2 cells were maintained in Dulbecco’s modified Eagle medium supplemented with 10% fetal bovine serum (FBS), 0.1 mM nonessential amino acids, and 1 mM sodium pyruvate. NCI-H23 and LU65 cells were maintained in Roswell Park Memorial Institute 1640 medium supplemented with 10% FBS. In all culture media, 100 U/mL penicillin and 100 μg/mL streptomycin were added to prevent bacterial contamination. All cell culture reagents were purchased from Gibco, Thermo Fisher Scientific (Waltham, MA, USA). The cell lines were incubated in a humidified incubator at 37 °C under 5% CO_2_ and 95% air atmosphere. All cells were verified through short-tandem repeat (STR) profiling and tested negative for mycoplasma contamination.

Sotorasib, ARS-1620, Q-VD-OPh (QVD), cobimetinib, N-acetyl-l-cysteine (NAC), and encorafenib were purchased from MedChemExpress (Monmouth Junction, NJ, USA).

### Establishment of sotorasib-resistant (SR) cells

We used a dose escalation approach reported in our previous study to establish sotorasib-resistant cells [15]. Briefly, the aforementioned parental cells were cultured at 50% confluence in the presence of sotorasib. The initial concentration of sotorasib was 0.1 μM; the concentration was increased by 0.1–0.3 μM every 2–3 days until the sotorasib concentration of 5 μM was attained. It took about 4–6 weeks to establish the sotorasib-resistant cells. The cells were cultured at this concentration until a stable cell proliferation rate was achieved. Before the experiments, we cultured sotorasib-resistant cells in the presence of 5 μM sotorasib for > 2 weeks to ensure that cells adapted to the presence of this KRAS^G12C^ inhibitor. Sotorasib-resistant cells were maintained in the same culture medium that was used for their parental cell, except that 5 μM sotorasib was added in the medium.

### Whole exome sequencing

The method of whole exome sequencing was described in our previous report [15]. The result was deposited to BioStudies ArrayExpress database under the accession numbers: E-MTAB-12144.

### Cell proliferation assay

To compare the proliferation rates between parental and resistant cells in the presence or absence of sotorasib, the cells were seeded at the density of 5 × 10^4^ cells/well in 6-well culture plates in triplicate for each condition. At the indicated time points, the cells were trypsinized, concentrated through centrifugation at 1000 rpm for 5 min, and resuspended in 20 μL of phosphate-buffered saline (PBS). The cells were stained with 20 μL of trypan blue solution [0.4% (w/v); Sigma-Aldrich, St. Louis, MO, USA] and were counted using an Invitrogen Countess 3 Automated Cell Counter (Thermo Fisher Scientific). To determine whether the inhibitors could rescue or aggravate the death of resistant cells upon sotorasib withdrawal, the cells were seeded at density of 2 × 10^3^ cells/well in 12-well culture plates in triplicate for each condition and were treated with different doses of the inhibitors for the indicated days, and cell numbers were counted as per the method described in the earlier text.

### Annexin V/propidium iodide (PI) flow cytometry assay

The cells were seeded at 50% confluency in 6-cm culture plates and treated for the indicated days, as presented in figure legends. On the day of the analysis, both the floating and attached cells were collected in 1 × binding buffer, resuspended and stained using the Annexin V-FITC Apoptosis Staining/Detection Kit (Abcam, Cambridge, UK). The samples were filtered through Falcon 5-mL Round Bottom Polystyrene Test Tubes (Corning, Corning, NY, USA) and analyzed on an LSR II flow cytometer (BD Biosciences, Franklin Lakes, NJ, USA); 1 × 10^4^ cells were counted for each sample. All data were analyzed using FlowJo (FlowJo LLC, Ashland, OR, USA).

### Caspase-3 activity assay

The Caspase-3/CPP32 Colorimetric Assay Kit was purchased from Abcam. The cells were seeded at 50% confluence in 10-cm culture plates in the presence or absence of sotorasib for the indicated days. On the day of the analysis, the cells were pelleted and lysed according to the manufacturer’s instruction. At least 150 μg of protein from each sample was used for this assay. Absorbance at 405 nm was measured on a Rayto RT-6900 Microplate Reader (Rayto, Shenzhen, China). The assay was performed in technical triplicate for each condition.

### Western blotting

Protein samples (20–50 μg per lane) were separated through 7–13% sodium dodecyl sulfate polyacrylamide gel electrophoresis and then electrotransferred onto BioTrace NT Membranes (Pall, Port Washington, NY, USA). These membranes were then incubated with primary and secondary antibodies at optimum dilutions, and immunoreactive signals were detected using an Immobilon Crescendo Western HRP substrate (Millipore Burlington, MA, USA) and MultiGel-21 (TOPBIO, New Taipei City, Taiwan). The antibodies used and their dilutions are listed in Additional file 1: Table S1.

### 5-Bromo-2′-deoxyuridine (BrdU) incorporation assay

The cells were seeded at 50% confluence in 6-cm culture plates in the presence or absence of sotorasib for the indicated days. On the day before analysis, the cells were incubated with 10 μM BrdU for approximately 16 h and evaluated using the FITC BrdU Flow Kit (BD Bioscience), according to the manufacturer’s instructions, on the next day. The samples were filtered through Falcon 5-mL Round Bottom Polystyrene Test Tubes and analyzed on an LSR II flow cytometer; 1 × 10^4^ cells were counted for each sample. The data were analyzed using FlowJo.

### CellROX staining

The cells were seeded at 50% confluence in 6-cm culture plates in the presence or absence of sotorasib for the indicated days. After treatment, CellROX Green Reagent (Thermo Fisher Scientific) was added in the medium to a final concentration of 5 μM and incubated for 30 min at 37 °C according to the manufacturer’s instructions. The cells were then harvested and filtered through Falcon 5-mL Round Bottom Polystyrene Test Tubes, followed by analysis using an LSR II flow cytometer; 1 × 10^4^ cells were counted for each sample. The data were analyzed using FlowJo.

### Immunofluorescence staining

After treatment, the cells were seeded at lower than 50% confluence in 6-well culture plates containing sterile coverslips before performing immunofluorescence. These coverslips were washed with PBS three times, fixed with 4% (v/v) paraformaldehyde/PBS for 5 min and permeabilized with 0.5% (v/v) Triton X-100/PBS. After incubating them in a blocking solution [5% bovine serum albumin (BSA) + 0.1% (v/v) Triton X-100/PBS] at room temperature for at least 1 h, the samples were then incubated with primary antibodies overnight at 4 °C. The cells were washed three times with 0.1% (v/v) Triton X-100/PBS and then incubated with secondary antibodies at room temperature for at least 1 h. For triple staining of phospho-histone variant H2A.X (S139) (γH2AX), p-histone H3 and cleaved caspase-3, the samples were incubated with all three relevant antibodies at room temperature for at least 1 h after blocking. The antibodies used and their dilutions are listed in Additional file 1: Table S2. Nuclei were stained using 1 μg/μL 4′,6′-diamino-2-phenylindole (DAPI). After they were stained, the coverslips were mounted onto microscope slides with DAKO Fluorescence Mounting Medium (Agilent, Santa Clara, CA, USA). After the slides were air-dried, images were acquired using an Olympus BX51 Fluorescence Microscope (Olympus, Tokyo, Japan) or a Leica TCS SP8 X Confocal Microscope (Leica Microsystems, Wetzlar, Germany). The images were analyzed using DP2-BSW software (Olympus) or LAS X Life Science Microscope software (Leica Microsystems), respectively.

### γH2AX quantification

The quantitation of γH2AX foci was performed manually to define DNA damage levels. For each of the three replicates in each condition, 200 nuclei were randomly selected. The cells with more than five γH2AX foci were denoted as “+” and those with pan-nuclear staining were defined as “ + + ”.

### 5-Ethynyl-2′-deoxyuridine (EdU) incorporation assay followed by immunofluorescence

The cells were seeded at 50% confluence in 6-well culture plates containing sterile coverslips. After treatment, the cells were incubated with 10 μM EdU. The incorporated EdU was detected using the Click-iT EdU Imaging Kits (Invitrogen, Thermo Fisher Scientific). Then, the cells were blocked using BSA and labeled using antibodies, according to the aforementioned immunofluorescence method. Nuclei were stained using Hoechst 33,342 provided in the kit, and the images were acquired using a Leica TCS SP8 X Confocal Microscope after the slides were mounted using DAKO Fluorescence Mounting Medium.

### Time-lapse in vitro microscopy and image analysis

The cells were seeded at 50% confluence in 6-well culture plates in the presence or absence of sotorasib for the indicated days and then cultured in the incubator of Axio Observer 7 (Carl ZEISS, Oberkochen, Germany) at 37 °C in a 5% CO_2_ and 95% air atmosphere. Images were acquired every 10 min for 16 h, followed by analysis using the ZEN software (Carl ZEISS).

### Comet assay

Comet assay was used to detect single- and double-stranded DNA breaks. In brief, the cells were harvested and resuspended in PBS at the density of 7.5 × 10^5^ cells/mL. After mixed with 500 μL of molten LMAagarose provided in the Comet Assay kit (R&D Systems, Minneapolis, MN, USA), cells were added in 50 μL of the mixture to a slide with a dry layer of agarose. For electrophoresis, the slides were immersed in 1 × Neutral Electrophoresis Buffer, and an electric current of 30 V was applied for 20 min. For DNA staining, 100 μL of dilute SYBR Gold (1:1000 in ddH_2_O; Thermo Fisher Scientific) was placed onto each dried agarose slide and stained for 30 min at room temperature in the dark. Images were taken using an Olympus BX51 Fluorescence Microscope.

### Xenograft experiments

The animal study was conducted in accordance with the guidelines of Council of Agriculture, Taiwan, and was approved by the Institutional Animal Care and Use Committee of Medical School, National Taiwan University (approval number 20210316). In total, 5 × 10^6^ cells were subcutaneously implanted into the flanks of 6-week-old female NOD/SCID mice (National Laboratory Animal Center, Taipei, Taiwan). To maintain an appropriate sotorasib concentration in the serum, sotorasib was orally administered at 10 mg/kg/day to all mice 1 day before injection and after injection until the mean tumor volume reached approximately 100 mm^3^. The mice were randomly assigned to four groups (n = 8 per group) that received vehicle (2% hydroxypropyl methylcellulose), sotorasib (10 mg/kg), encorafenib (20 mg/kg), or sotorasib (10 mg/kg) + encorafenib (20 mg/kg) through oral gavage daily. The mouse body weights and tumor sizes were monitored every 2 days. The tumors were measured using calipers, and tumor volume was calculated using the following formula: 1/2 × [length (mm) × width^2^ (mm^2^)]. After 14 days of treatment, the mice were sacrificed, and their tumors were collected and weighed.

### Statistical analysis

The results were analyzed using GraphPad Prism 9.5.1 (San Diego, CA, USA). Data were first tested for normality using Shapiro’s test. For a comparison of two groups, two-tailed unpaired Student’s t test was used. For a comparison of multiple groups, the statistical analyses included one-way or two-way ANOVA, followed by post-hoc Tukey testing of pairwise comparisons. Chi-squared tests were used for frequency distributions. Significance was established at *P* < 0.05.

## Results

### Effects of sotorasib withdrawal on sotorasib-resistant KRAS^G12C^-mutant cancer cells

**Fig. 1:**
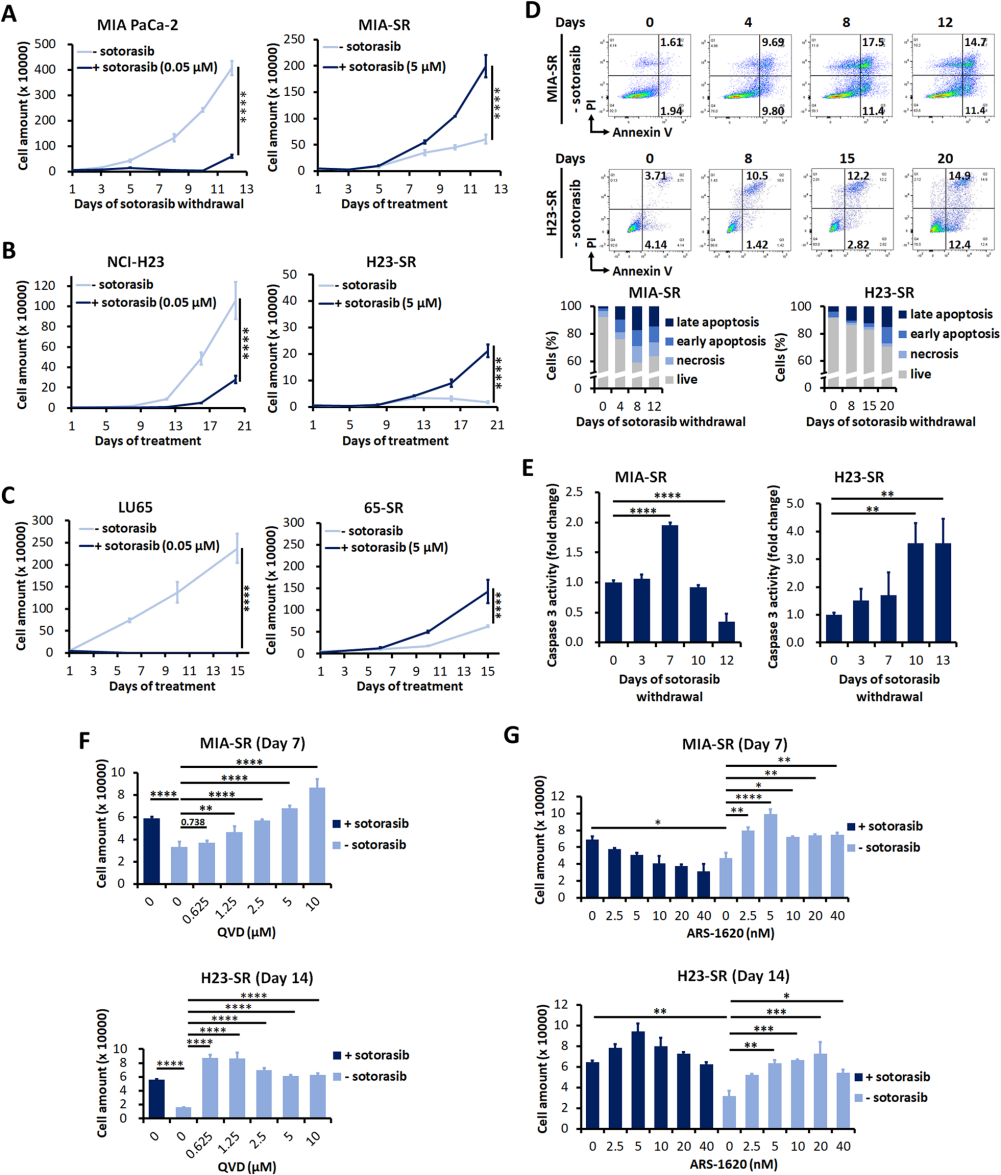
Withdrawal of KRAS^G12C^ inhibitor results in the death of sotorasib-resistant KRAS^G12C^-mutant cancer cell. **A–C** Growth curves of MIA-PaCa-2, NCI-H23, and LU65 cells and their sotorasib-resistant sublines MIA-SR, H23-SR, and 65-SR cultured with (+) or without (−) of 5 μM sotorasib. **D, E** Results of (**D)** annexin V/PI flow cytometry assay and (**E**) caspase-3 activity assay performed using the MIA-SR and H23-SR after sotorasib withdrawal. **F** The MIA-SR and H23-SR cells were treated with different concentrations of the caspase inhibitor QVD after sotorasib withdrawal; the cells were counted on Day 7 (MIA-SR) and 14 (H23-SR). **G** The MIA-SR and H23-SR cells were cultured with or without 5 μM sotorasib in the presence of different concentrations of ARS-1620; the cell numbers were counted on Day 7 (MIA-SR) and 14 (H23-SR). Data (in **A–C**, **E–G**) are presented in terms of mean ± standard deviation values of three replicates. *P < 0.05, **P < 0.01, ***P < 0.001, and ****P < 0.0001

To analyze the responses of sotorasib-resistant KRAS^G12C^-mutant cancer cells to drug withdrawal, we attempted to generated sotorasib-resistant cancer cells by exposing to sequentially increasing doses of sotorasib from the starting concentration of 0.1–5 μM for 4–6 weeks. SW1463 and H358 cells could not tolerate sotorasib at a dose higher than 0.2 μM; thus, resistant SW1463 and H358 cells could not be established. The treatment naive LU99 cells were sotorasib resistant (IC_50_: 22.55 μM). Hence, it was excluded for further experiments. Finally, sotorasib-resistant MIA PaCa-2, NCI-H23, and LU65 cells was established (hereafter, referred to as MIA-SR, H23-SR, and 65-SR, respectively). Short tandem repeat profiling confirmed the identity of SR cells to their parental cells (Additional file 1: Table S3–S5). Whole exome sequencing didn’t identify difference of single nucleotide variant and copy number variation in genes related to cell proliferation, signal transduction and drug metabolism between treatment naive and sotorasib-resistant cells, indicating the resistance was through nongenetic mechanisms (Additional file 1: Table S6). All the three sotorasib-resistant cells grew actively in the presence of sotorasib (Additional file 1: Fig. S1). However, intriguingly, the MIA-SR cells cultured in the sotorasib-free medium demonstrated a significantly smaller cell number increase rate than did those cultured in the sotorasib-containing medium (Fig. 1A). Furthermore, sotorasib withdrawal caused gradual reduction in H23-SR cell numbers from days 12 to 20 (Fig. 1B). Although the MIA-SR and H23-SR cells displayed this “sotorasib addiction” phenomenon, the 65-SR cells showed reductions in the growth rate until day 10, but thereafter, this rate became similar to that of the 65-SR cells cultured in the sotorasib-containing medium (Fig. 1C; Additional file 1: Fig. S2). We performed an annexin V/PI flow cytometry assay to investigate whether sotorasib addiction occurred in the MIA-SR and H23-SR cells because of cell death and to identify the mechanisms underlying cell death. Increased dead cell numbers were noted for both the MIA-SR and H23-SR cells after sotorasib withdrawal; the dead cells were mainly in the annexin V^+^/PI^+^ compartment (Fig. 1D). Moreover, after 7 days of withdrawal, caspase-3 activity was two times higher in the MIA-SR cells than in the MIA-SR cells cultured in the sotorasib-containing medium (Fig. 1E). Similar results were observed for the H23-SR cells, but with a higher fold change than with that of the MIA-SR cells—consistent with the more evident decrease in the H23-SR cell number than in the MIA-SR cell number after sotorasib withdrawal (Fig. 1A and B). The pan-caspase inhibitor QVD counteracted the cell death due to sotorasib withdrawal (Fig. 1F). These results indicated that caspase-dependent apoptosis may be a mechanism underlying sotorasib dependence. To prove that the observed effects were due to the on-target effects of sotorasib, ARS-1620, a structurally distinct KRAS^G12C^ inhibitor [8], was added, and the results revealed that the addition of ARS-1620 rescued sotorasib withdrawal–induced cell death (Fig. 1G). Taken together, these results indicate that sotorasib-resistant cancer cells may undergo cell death after KRAS^G12C^ inhibitor deprivation, and that caspase-dependent apoptosis is a mechanism underlying cell death.

### MAPK pathway hyperactivation is the mechanism for death of sotorasib-resistant cells after sotorasib withdrawal

**Fig. 2:**
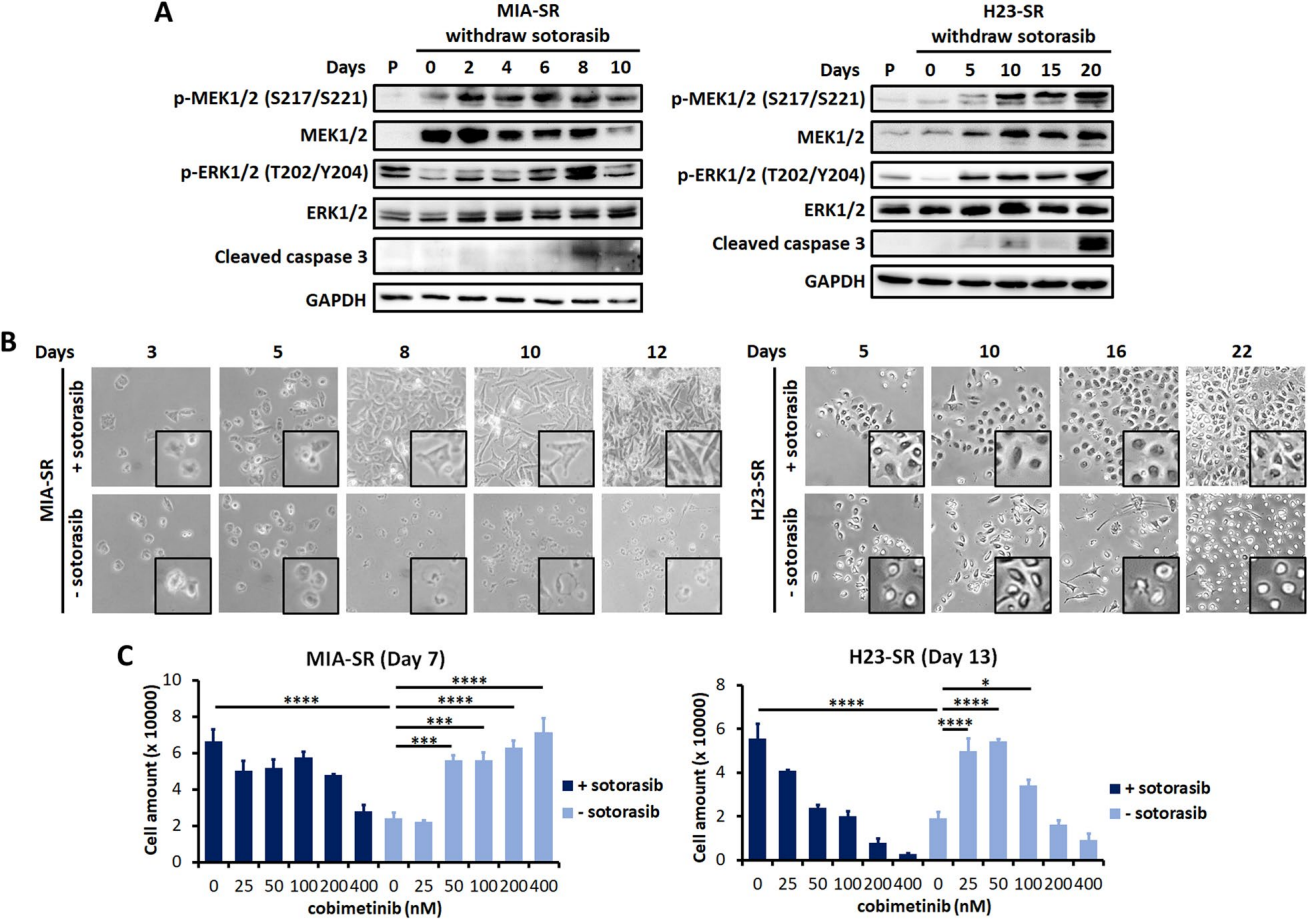
Hyperactivity of the MAPK signaling pathway causes the sotorasib-addiction phenotype.** A** Effects of sotorasib withdrawal on the level of p-MEK1/2, MEK, p-ERK, ERK, and cleaved caspase-3 in the MIA-SR and H23-SR cells; protein levels were measured through Western blotting. **B** Phase-contrast microscopy images of the MIA-SR and H23-SR cells cultured with or without 5 μM sotorasib. **C** The MIA-SR and H23-SR cells were cultured with or without 5 μM sotorasib in the presence of different concentrations of the MEK inhibitor cobimetinib. The cells were counted on Day 7 (MIA-SR) and 13 (H23-SR). Data are presented in terms of mean ± standard deviation values of three replicates. *P < 0.05, ***P < 0.001, and ****P < 0.0001

The MAPK pathway is the primary downstream pathway activated by KRAS [6]. Therefore, we determined the effects of sotorasib withdrawal on MAPK signaling. The MIA-SR cells exhibited higher basal levels of MEK1/2 and higher levels of active MEK1/2 than did the parental cells (Fig. 2A); however, the activation of the downstream effector extracellular-signal-regulated kinase 1/2 (ERK1/2) was constrained by sotorasib before withdrawal. The levels of both p-MEK1/2 and p-ERK1/2 increased after sotorasib withdrawal; after 8 days of sotorasib withdrawal, the phosphorylation level decreased. Cleaved caspase-3 was detected starting from day 8 of sotorasib withdrawal—consistent with the reduction of the cell number increase rate (Fig. 1A) and the increase in caspase-3 activity (Fig. 1E). Similar results were observed for the H23-SR cells, except for MEK/ERK activation rebounding after longer days of sotorasib withdrawal and persisted more days than that for the MIA-SR cells did, probably due to the much slower growth rate of the H23-SR cells.

A hyperactivated RAS/MEK/ERK cascade can induce myosin-dependent changes in the cell shape during the interphase, with the cells adopting a rounded, sphere-like morphology [21, 22]. Consistent with these reports, we observed that the morphology of sotorasib-resistant cells gradually transformed into a rounded shape with loss of cell–cell contact after sotorasib withdrawal; this was in contrast to the flat and adherent morphology noted in the presence of sotorasib (Fig. 2B).

Although the MAPK pathway is essential for cell proliferation and neoplastic transformation, untimely or excessive MAPK pathway activation can paradoxically induce cell death [23, 24]. Here, the MEK inhibitor cobimetinib counteracted the cell death driven by sotorasib withdrawal (Fig. 2C). Taken together, these data demonstrated that the excessive, continuous rebound of the MAPK pathway after sotorasib withdrawal is responsible for the death of sotorasib-resistant cells cultured without sotorasib.

### Sotorasib withdrawal induces DNA damage and mitotic catastrophe in sotorasib-resistant cells

**Fig. 3:**
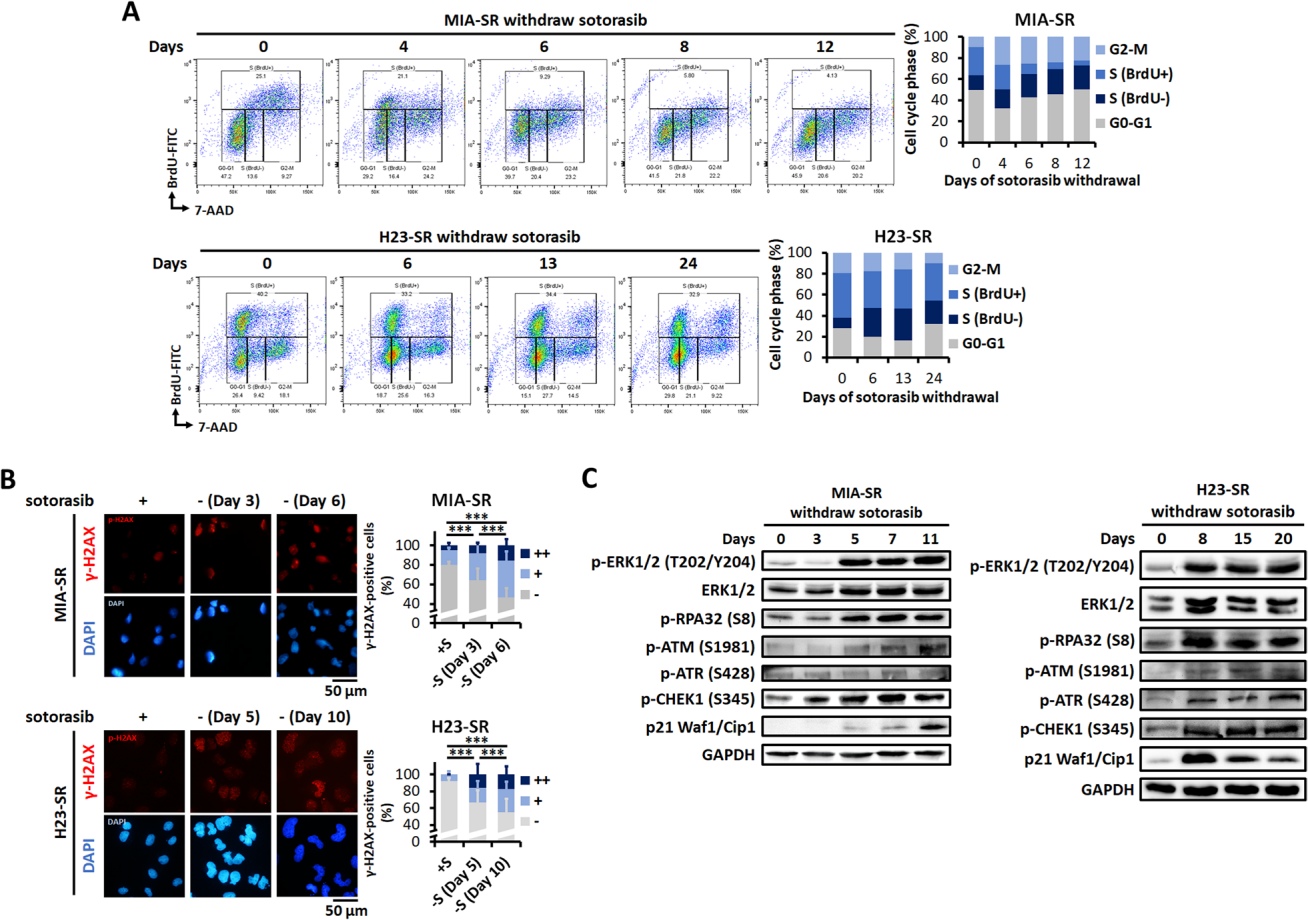
Sotorasib withdrawal induces DNA damage and replication stress.** A** DNA synthesis and cell cycle distribution assessed through the BrdU incorporation assay in the MIA-SR and H23-SR cells cultured with or without 5 μM sotorasib for the indicated days. **B** Representative images of immunofluorescence staining and quantification of γ-H2AX staining pattern performed using the MIA-SR and H23-SR cells cultured with or without 5 μM sotorasib for the indicated days. The cell nuclei were visualized using DAPI (blue). A minimum of 200 nuclei per condition were analyzed. −: < 5 foci/cell, +: > 5 foci/cell, and ++ : pan-nuclear expression. **C** Effects of sotorasib withdrawal on DNA damage checkpoint proteins in the MIA-SR and H23-SR cells; protein levels were measured through Western blotting. ***P < 0.001

We conducted BrdU incorporation assay to study the effects of sotorasib withdrawal on cell cycle progression. As illustrated in Fig. 3A, sotorasib withdrawal induced a considerable reduction in BrdU incorporation in the S phase and increased the fraction of G2/M cells in the MIA-SR cells, indicating defects in the S phase and G2/M progression. In the H23-SR cells, sotorasib withdrawal induced an increase in the fraction of BrdU(-) cells in the S phase, indicating defective in DNA synthesis during S phase.

Next, we investigated the mechanisms underlying cell cycle arrest and cell death in sotorasib-resistant cells after sotorasib withdrawal. Increased reactive oxidative species (ROS) production, enhanced macropinocytosis (methuosis), and DNA damage were the mechanisms proposed for MAPK pathway-induced cell death [17, 23, 24]. However, CellROX staining demonstrated increased total ROS levels in the MIA-SR cells, but not in the H23-SR and 65-SR cells, after sotorasib withdrawal (Additional file 1: Fig. S3A). NAC, an ROS scavenger, failed to rescue death of sotorasib-resistant cells after sotorasib withdrawal (Additional file 1: Fig. S3B). We did not observe increased pinocytic vesicle formation after sotorasib withdrawal (Additional file 1: Fig. S4). Therefore, increased ROS production and enhanced macropinocytosis were not the major mechanism underlying sotorasib withdrawal–induced cell death.

To analyze the effects of sotorasib withdrawal on genome integrity, we performed immunofluorescent staining for γ-H2AX. The staining patterns were classified into three categories: less damaged (referred to as “−” for < 5 foci/cell), more damaged (referred as “+” for > 5 foci/cell) and replication stress–associated levels (referred as “ + + ” for pan-nuclear expression). A significant increase in the percentage of cells with a high number of γ-H2AX foci and pan-nuclear staining was observed in the MIA-SR and H23-SR cells after sotorasib withdrawal (Fig. 3B). We next determined whether DDR was activated by sotorasib withdrawal. An early response to replication stress is replication protein A (RPA) accumulated on single-stranded DNA, which recruits and activates ataxia telangiectasia and Rad3-related protein (ATR). Activated ATR phosphorylates many targets, including the RPA32 subunit of RPA, leading to checkpoint kinase 1 (CHEK1) activation and replication arrest [25]. After sotorasib withdrawal, p-ATM (ataxia-telangiectasia mutated kinase), p-ATR, p-RPA32, and p-CHEK1 levels gradually increased (Fig. 3C). Thus, from approximately 5 days after withdrawal, p21 levels increased, decelerating the cell cycle. These results indicated that in the absence of suppression by sotorasib, sustained MEK/ERK signaling induced DNA damage and triggered DDR activation, resulting in cell cycle arrest in sotorasib-resistant cells.

**Fig. 4:**
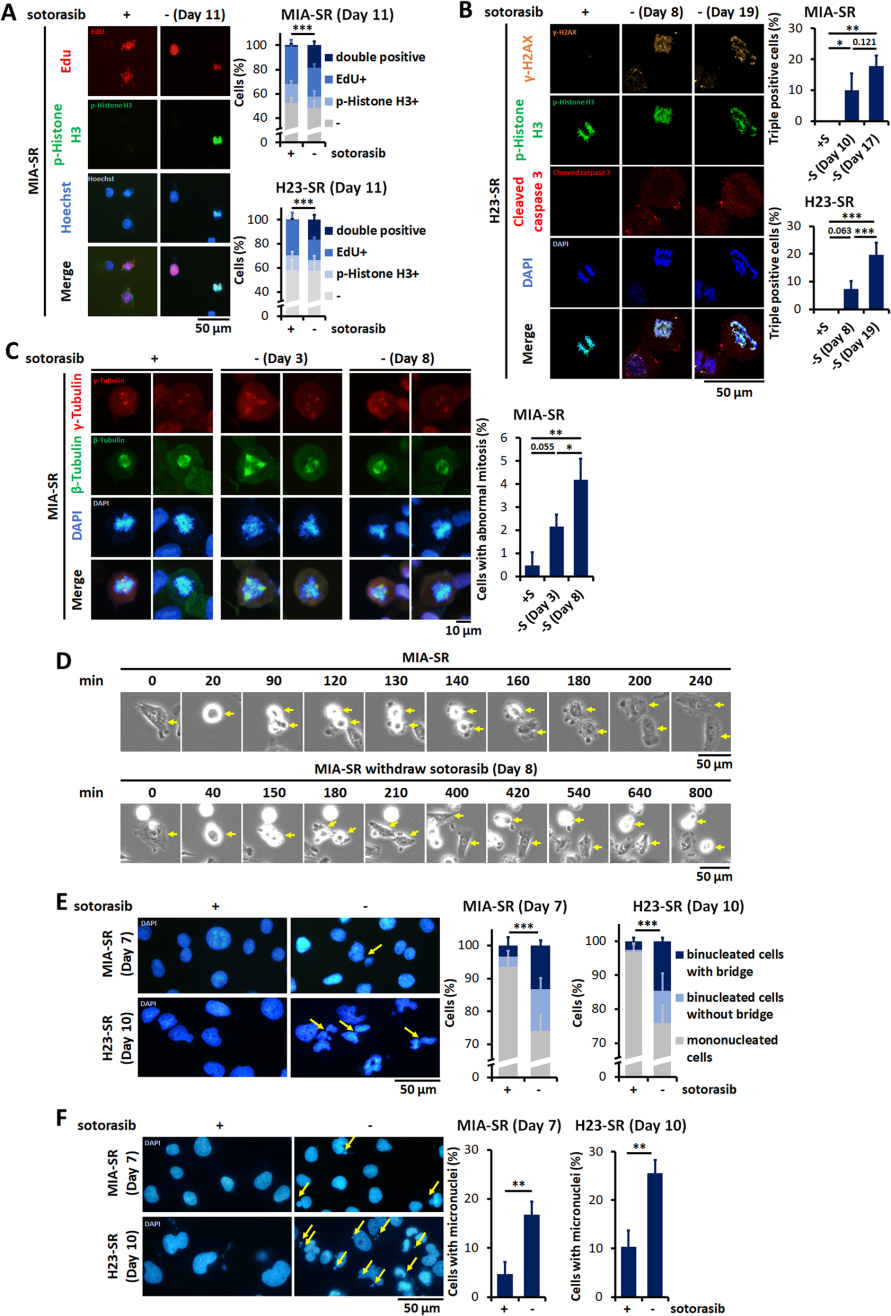
Sotorasib withdrawal induces premature mitotic entry and aberrant mitosis. **A** The MIA-SR and H23-SR cells were cultured with or without 5 μM sotorasib for 11 days and pulsed with iFluor 594-labeled EdU (red) for 4 h. p-Histone H3 (green) was detected through immunofluorescence staining. **B** The MIA-SR and H23-SR cells were cultured with or without 5 μM sotorasib for the indicated days and then subjected to immunofluorescence staining with γ-H2AX (orange), p-histone H3 (green), and cleaved caspase-3 (red) antibodies. **C** Representative images of immunofluorescence staining of the MIA-SR cells in the mitotic phase. The MIR-SR cells were cultured with or without 5 μM sotorasib for the indicated days and then stained with γ-tubulin (red) and β-tubulin (green). The quantification of abnormal mitosis was defined as more than one pair of centrosomes (γ-tubulin +) and mitotic spindles (β-tubulin +) in one cell. **D** Time-lapse microscopy images of the MIA-SR cells cultured with or without 5 μM sotorasib for the indicated days. The yellow arrows denote the indicated cell. **E, F** Representative images of immunofluorescence staining and quantification of nucleoplasmic bridges (**E**) and micronuclei (**F**) in the MIA-SR and H23-SR cells cultured with or without 5 μM sotorasib. The yellow arrows denote the locations of nucleoplasmic bridges (**E**) or micronuclei (**F**). A minimum of 200 nuclei per condition were analyzed (**A**–**C, E, F).** *P < 0.05, **P < 0.01, and ***P < 0.001

In cells with extensive DNA damage, RPA is insufficient for mediating the repair of an excessive amount of single-stranded DNA, and the collapsed replication forks may lead to double-stranded DNA breaks (DSBs). This prolonged replication stress along with exhausted repair mechanisms can trigger mitotic catastrophe when cells enter mitosis without their DSBs being repaired [26]. EdU–phospho-histone H3 coexpression was noted in the sotorasib-resistant cells after sotorasib withdrawal (Fig. 4A), indicating that the sotorasib-resistant cells proceeded to mitosis with an incompletely replicated genome. To confirm that sotorasib withdrawal induces mitotic catastrophe in sotorasib-resistant cells, the cells were stained with γ-H2AX, phospho-histone H3 and cleaved caspase-3. We detected frequent coexpression of these three markers in the sotorasib-resistant cells after sotorasib withdrawal, which is a marker of mitotic catastrophe (Fig. 4B) [27]. Abnormal mitotic figures were also frequently detected in the withdrawal group (Fig. 4C). Live-cell imaging using time-lapse microscopy demonstrate prolonged cycling time during mitosis and frequent death of daughter cells (Fig. 4D, Additional file 1: Fig. S5, Additional file 2: Movie 1, Additional file 3: Movie 2, Additional file 4: Movie 3, Additional file 5: Movie 4). As a consequence of mitotic catastrophe, increased numbers of cells with micronuclei and multilobulated nuclei with nucleoplasmic bridges were observed in the sotorasib-resistant cells after sotorasib withdrawal (Fig. 4E and F). Taken together, these results indicate that sotorasib withdrawal causes the premature entry of cells with damaged DNA into the M phase and in turn leads to abnormal mitosis and caspase-dependent death in sotorasib-resistant cells.

**Fig. 5:**
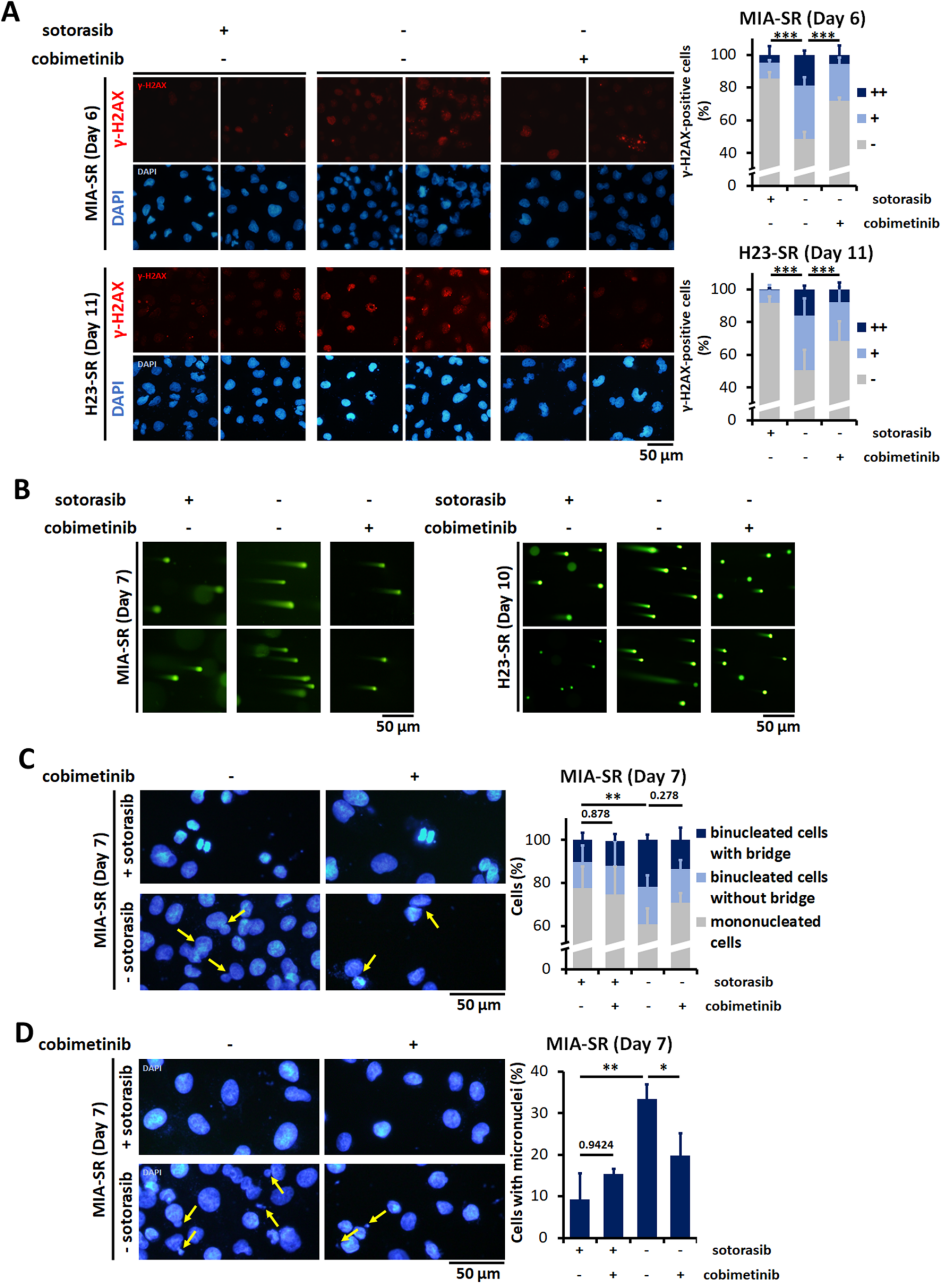
Inhibition of MAPK pathway prevents DNA damage and reverses nuclear abnormality in sotorasib-resistant cells.** A** The MIA-SR and H23-SR was cultured with or without 5 μM sotorasib, 200 nM cobimetinib, or both. γ-H2AX (red) was detected through immunofluorescence. −: < 5 foci/cell, +: > 5 foci/cell, and + + : pan-nuclear expression. **B** The MIA-SR and H23-SR cells were cultured with or without 5 μM sotorasib, 200 nM cobimetinib, or both. The degree of DNA strand breaks was measured through comet assay. **C, D** Representative images of immunofluorescence staining and quantification of nucleoplasmic bridges (**C**) and micronuclei (**D**) in the MIA-SR cells cultured with or without sotorasib, 200 nM cobimetinib, or both. The yellow arrows denote the locations of nucleoplasmic bridges (**C**) or micronuclei (**D**). A minimum of 200 nuclei per condition were analyzed (**A**, **C**, **D**). *P < 0.05, **P < 0.01, ***P < 0.001, and ****P < 0.0001

We next explored whether replication stress and mitotic catastrophe induced by sotorasib withdrawal are due to MAPK pathway hyperactivation. Inhibiting the MAPK signaling pathway with cobimetinib considerably reduced the numbers of γ-H2AX-positive cells and the numbers of cells with pan-nuclear staining (Fig. 5A). Comet assay results demonstrated that DNA damage induced by sotorasib withdrawal was rescued by cobimetinib (Fig. 5B). Cobimetinib treatment also prevented abnormal mitosis processes in the sotorasib-resistant cells, resulting in the normalization of the numbers of cells with micronuclei and nucleoplasmic bridging (Fig. 5C and D).

In summary, after sotorasib withdrawal, the sotorasib-resistant cells demonstrated DNA damage driven by sustained MAPK activity, inducing replication stress and initiating DDR-mediated repair mechanisms. However, the excessive and prolonged damage forced the incompletely repaired cells to prematurely progress into the M phase, resulting in abnormal mitosis, daughter cells with an abnormal genome and mitotic cell death.

### Aggravation of post-sotorasib-withdrawal DNA damage and death in sotorasib-resistant cells by low-dose BRAF inhibitor treatment

**Fig. 6:**
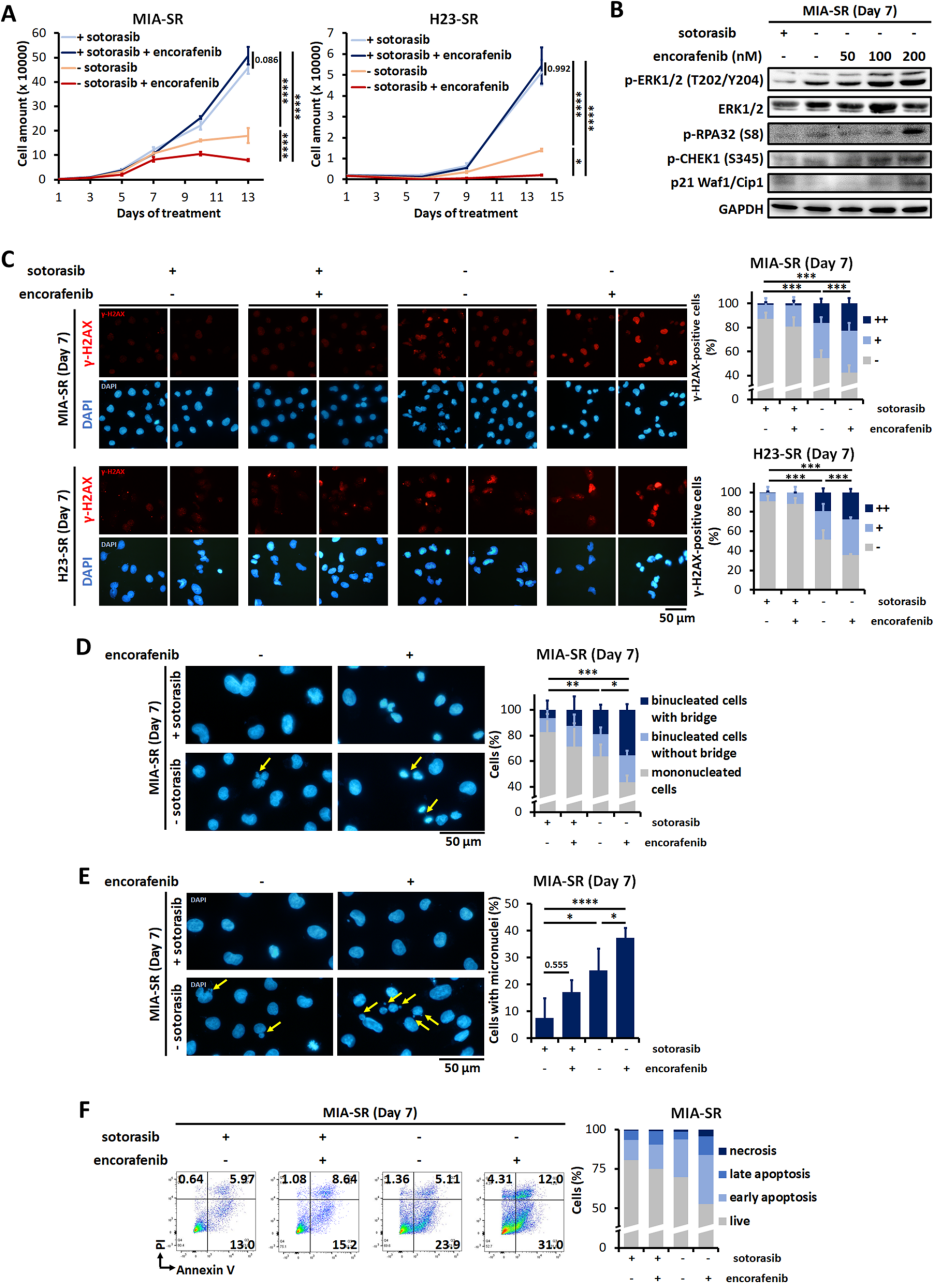
Low-dose BRAF inhibitor aggravates sotorasib withdrawal–induced DNA damage and cell death. **A **The MIA-SR and H23-SR cells were cultured with or without 5 μM sotorasib, 100 nM encorafenib, or both. The cells were counted on the indicated days. Data are presented in terms of mean ± standard deviation values of three cell culture replicates. **B** Effects of encorafenib treatment on the level of p-ERK, ERK, p-RPA32, p-CHEK1, and p21 in the MIA-SR cells cultured with or without 5 μM sotorasib; protein levels were measured through Western blotting. **C** Representative images of immunofluorescence staining and quantification of γ-H2AX staining pattern performed using the MIA-SR and H23-SR cells cultured with or without 5 μM sotorasib, 100 nM encorafenib, or both for the indicated days. −: < 5 foci/cell, +: > 5 foci/cell, and + + : pan-nuclear expression. **D, E** Representative images of immunofluorescence staining and quantification of nucleoplasmic bridges (**D**) and micronuclei (**E**) in the MIA-SR cells cultured with or without 5 μM sotorasib, 100 nM encorafenib, or both. The yellow arrows indicate the locations of micronuclei (**D**) or nucleoplasmic bridges (**E**).** F** Results of annexin V/PI flow cytometric assay performing using the MIA-SR cells cultured with or without 5 μM sotorasib, 100 nM encorafenib, or both. A minimum of 200 nuclei per condition were analyzed (**C**–**E**). *P < 0.05, **P < 0.01, ***P < 0.001, and ****P < 0.0001

Because death in the sotorasib-addicted cells was noted to be induced through MAPK cascade hyperactivation and DNA damage, it prompted us to test whether deliberately enhancing MAPK signaling aggravates sotorasib withdrawal–induced cell death. Type I RAF inhibitors can paradoxically activate the MAPK pathway in mutant *KRAS* but wild-type *BRAF* tumors [28]. Therefore, we treated the MIA-SR and H23-SR cells with the BRAF inhibitor encorafenib. In the presence of sotorasib, the addition of encorafenib did not affect tumor cell viability. Compared with sotorasib withdrawal alone, low-dose encorafenib slowed down cell number increase more effectively in both the MIA-SR and H23-SR cells (Fig. 6A). Encorafenib treatment induced dose-dependent ERK activation in the MIA-SR and H23-SR cells after sotorasib withdrawal, resulting in the phosphorylation of RPA32 and CHEK1 as well as the induction of p21 (Fig. 6B and Additional file 1: Fig. S6). Consistent with the enhanced ERK activation, DNA damage, micronuclei formation, nucleoplasmic bridges, and apoptosis were induced by sotorasib withdrawal, which was further aggravated by encorafenib treatment (Fig. 6C–F and Additional file 1: Fig. S7–S8). Thus, pharmacologically augmenting MAPK pathway activation with BRAF inhibitor can aggravate DNA damage and cell death during sotorasib withdrawal.

### BRAF inhibitor counteracts the tolerance of 65-SR cells to sotorasib withdrawal

**Fig. 7:**
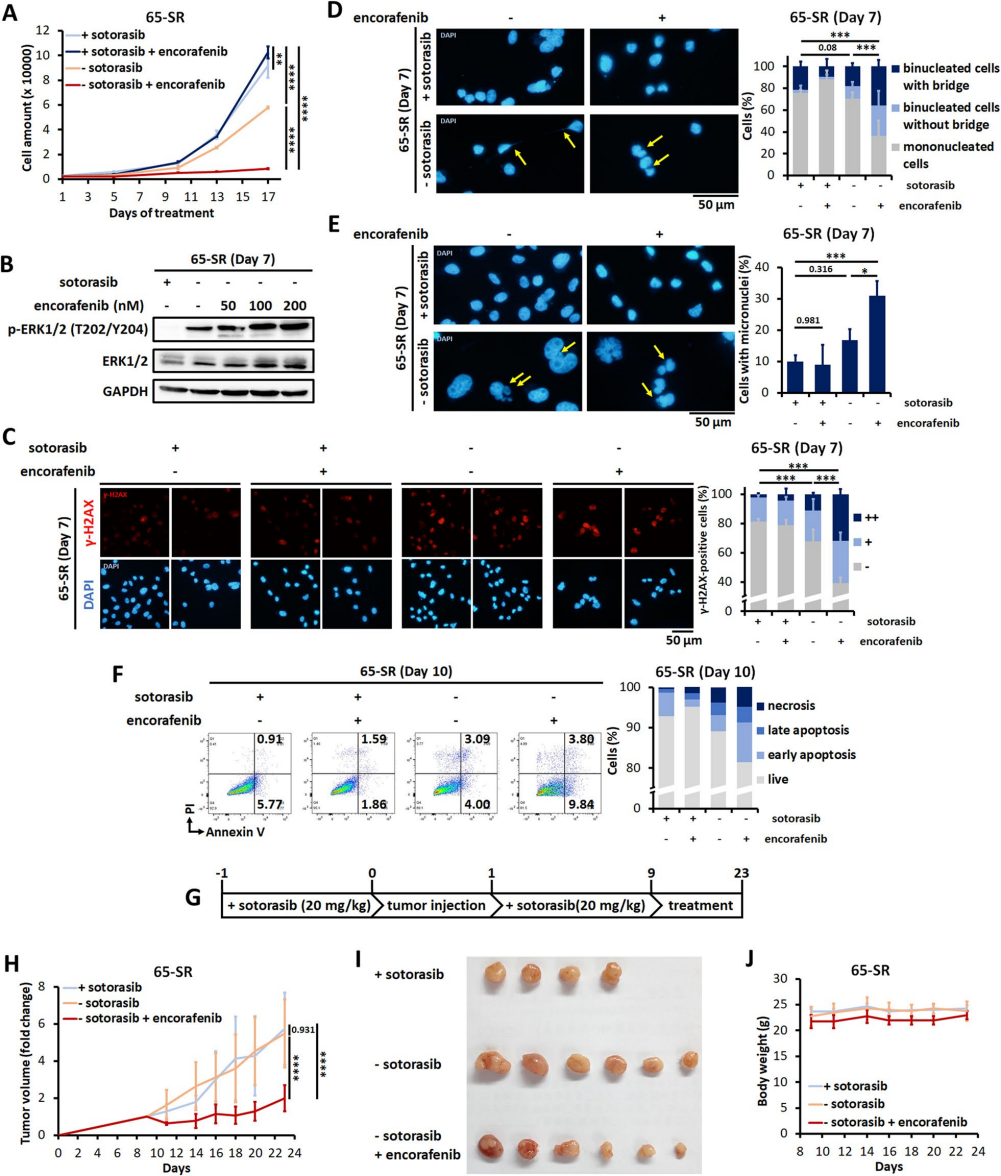
BRAF inhibitor sensitizes the 65-SR cells to sotorasib withdrawal. **A** The 65-SR cells were cultured with or without 5 μM sotorasib, 100 nM encorafenib, or both. The cells were counted on the indicated days. **B** Effects of encorafenib on the levels of p-ERK and ERK in the 65-SR cells cultured with or without 5 μM sotorasib; protein levels were measured through Western blotting. **C** Representative images of immunofluorescence staining and quantification of γ-H2AX staining pattern in the 65-SR with or without 5 μM sotorasib and/or 100 nM encorafenib for the indicated days. −: < 5 foci/cell, +: > 5 foci/cell, and ++ : pan-nuclear expression. **D, E** Representative images of immunofluorescence staining and quantification of nucleoplasmic bridges (**D**) and micronuclei (**E**) in the 65-SR cells cultured with or without 5 μM sotorasib, 100 nM encorafenib, or both. The yellow arrows denote the locations of nucleoplasmic bridges (**D**) or micronuclei (**E**). **F** Results of annexin V/PI flow cytometric assay on the 65-SR cells cultured with or without 5 μM sotorasib, 100 nM encorafenib, or both. **G** Schematic of the drug treatment of the 65-SR xenografts models. Sotorasib (20 mg/kg) was orally administered one day before and after tumor injection until the mean tumor volume reached at least 100 mm^3^. Next, the mice were divided into sotorasib +/encorafenib −, sotorasib-/encorafenib −, and sotorasib −/encorafenib + groups (n = 4–6 mice per group) and treated with the indicated drugs for 14 days. The concentration of encorafenib used was 20 mg/kg. **H–J** Tumor sizes (**H**) and body weights (**J**) of the mice were measured every 2 days. **I** At the end of experiment, the tumors were removed and photographed. Data are presented in terms of the mean ± standard deviation values of three cell culture replicates (**A**) or 4–6 mice (**H**, **J**). *P < 0.05, **P < 0.01, ***P < 0.001, and ****P < 0.0001

In contrast to the MIA-SR and H23-SR cells, the 65-SR cells survived after sotorasib withdrawal and grew considerably after day 10 despite a strong increase in MEK/ERK phosphorylation (Additional file 1: Fig. S9). We analyzed whether encorafenib treatment further augments MAPK pathway activity and converts the 65-SR cells from being withdrawal-resistant to being withdrawal-sensitive. Notably, the proliferation rate of the 65-SR cells was considerably suppressed by encorafenib treatment than that of the sotorasib withdrawal only or the sotorasib-containing groups (Fig. 7A). p-ERK1/2 levels increased after encorafenib treatment (Fig. 7B). Encorafenib treatment strongly increased the number of γ-H2AX-positive cells (Fig. 7C), resulting in more cells with micronuclei and nucleoplasmic bridging (Fig. 7D and E). Finally, the encorafenib-induced paradoxically activated MAPK pathway also augmented death in the 65-SR cells after sotorasib withdrawal (Fig. 7F).

### Combination of sotorasib withdrawal and BRAF inhibitor induces tumor regression in vivo

We finally assessed whether the effects of sotorasib addiction in sotorasib-resistant cells could be applied for treatment in vivo. In contrast to treatment-naive MIA-PaCa2 cells, the MIA-SR cells can’t generate stable subcutaneous xenograft, even the mice are treated with sotorasib (20 or 30 mg/kg per day) from three days before tumor cell injection. Hence, we implanted the 65-SR cells into the flanks of mice and tested the effects of encorafenib treatment with or without sotorasib (Fig. 7G and H). In the presence of sotorasib, encorafenib treatment demonstrated no effects on tumor size and mouse body weight (Additional file 1: Fig. S10). Consistent with our in vitro results, sotorasib withdrawal demonstrated no significant effects on the 65-SR tumor growth (Fig. 7H). However, encorafenib treatment during sotorasib withdrawal impaired tumor growth (Fig. 7H and I). The mouse body weights were maintained, indicating little toxicity to mice by encorafenib treatment (Fig. 7J). Taking together, these results indicate that robust MAPK pathway activation induced by sotorasib withdrawal and a type I BRAF inhibitor leads to conversion of the non–sotorasib-addicted xenografts to sotorasib-addicted xenografts.

## Discussion

Clinical studies have demonstrated that covalent inhibitors that selectively target KRAS^G12C^ have promising results against cancers harboring the *KRAS*^*G12C*^ mutation [10]; however, their treatment responses are limited by drug resistance development. In this study, we observed that the dependence of sotorasib-resistant tumors on the continuous presence of the drug resulted in fitness deficit after sotorasib deprivation. The drug addiction phenomenon is mediated by MAPK pathway hyperactivation, eventually triggering severe DNA damage, replication stress, and mitotic catastrophe.

The drug addiction phenomenon is well-documented in tumors resistant to BRAF or MAPK inhibitors. Although the MAPK pathway is a crucial for tumorigenesis, untimely or excessive activation of the MAPK pathway is deleterious to cancer cells [23, 24, 29, 30]. Therefore, cancers with mutation in the MAPK pathway must restrain the activity of ERK1/2 to avoid toxicities and enable tumor growth. In cells adopted to MAPK pathway inhibitors, drug removal frequently results in cell growth arrest or death, a phenomenon known as drug addiction. Addiction to MEK inhibitor has been demonstrated in trametinib-resistant lung adenocarcinoma cell lines through genetic alternations in the MAPK signaling pathway leads to hyperactivation of ERK2 and apoptosis after trametinib withdrawal [31]. In MEK inhibitor–resistant colorectal cell lines, selumetinib withdrawal results in cell cycle arrest and senescence through p57^KIP2^ induction and cell death through NOXA induction and BH3 interacting-domain death agonist (BID) cleavage to its activated form [32]. In MAPK inhibitor–resistant melanoma cell lines, MAPK inhibitor withdrawal causes considerable increases in mitochondrial ROS levels as well as mitochondrial swelling and depolarization. Mitochondrial dysfunction and depolarization induce apoptosis-inducing factor (AIF) cleavage and nuclear translocation, evoking parthanatos, a form of poly (ADP-ribose) polymerase 1 (PARP-1)-dependent programmed cell death [17]. Mutant KRAS has been noted to induce mitochondrial ROS production [33]. Therefore, in the current study, we first determined the effects of sotorasib withdrawal on ROS production. However, our results indicated that KRAS^G12C^ inhibitor deprivation did not induce excessive ROS production in all sotorasib-resistant cell lines, and the ROS scavenger NAC could not reverse DNA damage and cell death due to sotorasib withdrawal. Therefore, we explored other possible underlying mechanisms.

Oncogenic RAS can cause DNA damage through multiple mechanisms, including ROS production, alterations in replication forks, depletion of nucleotide pool, and transcription-replication collision [34]. Expression of HRas^V12^ in normal cells resulting in a hyper-replication state with increased origin firing, alterations in DNA replication fork progression, asymmetric replication fork generation, and cell division cycle 6 (CDC6) overexpression [35]. Mutant KRAS downregulates the expression of ribonucleotide reductase subunit M2 downregulation, causing depletion of deoxyribonucleotide triphosphate levels and DDR [36]. HRAS^V12^ expression induces elevated level of the general transcription factor TATA-box binding protein, leading to increased RNA synthesis, which together with R-loop accumulation results in replication fork slowing and DNA damage [37]. Mutant RAS also abrogates the DNA damage response. The cells with unrepaired DNA continued through mitosis resulting in defects in chromosome segregation and other forms of chromosome damage [38]. Consistent with these observations, the rapid activation of mutant RAS has been reported to induce replication stress and chromosome instability through the MAPK pathway in multiple cell models [38–40]. However, the details of molecular mechanisms linking MAPK pathway hyperactivation, DNA damage, and aberrant mitosis remain unclear. In the present study, we noted that robust MAPK pathway activation induced by sotorasib withdrawal in our sotorasib-resistant cells resulted in massive level of DNA damage and p21-mediated cell cycle arrest. Extensive DNA damage can cause exhausted DNA repair, resulting in the premature entry of the cells with unrepaired DNA into mitosis and eventually driving the cells toward mitotic catastrophe. Hence, although the drug addiction phenomenon was noted in KRAS, BRAF, and MEK inhibitor-treated cancers, the underlying mechanisms are different.

Drug holiday—a strategy of programmed treatment interruptions—is typically used to limit toxicity related to cancer therapy [41]. Recently, intermittent treatment has been proposed as a method to delay resistance onset, particularly in cancer therapy targeting MAPK [20, 42, 43]. Particularly in RAS/RAF-mutant tumors, the elevated baseline of MAPK signaling and the emergence of additional mutation render uncontrolled ERK activation lethal upon the removal of the negative regulator [17–20]. Therefore, the differences in cell fitness between drug treatment and holiday in a MAPK pathway–targeting treatment may be useful for inhibiting tumor cell proliferation by modulating MAPK pathway activity.

In this study, not all sotorasib-resistant cancer cell lines exhibited the drug addiction phenomenon. Because the treatment-resistant cell populations were heterogeneous, we anticipated that simply withdrawing sotorasib treatment may not induce tumor regression in clinical practice. Therefore, the development of novel strategies for enhancing the effects of sotorasib addiction is warranted. In line with the paradoxical activation of the MAPK pathway by type I BRAF inhibitors [28], we found that the addition of encorafenib enhanced the effects of sotorasib withdrawal and converted withdrawal-resistant cancer cells to withdrawal-sensitive cancer cells. Therefore, the combination of encorafenib treatment with sotorasib withdrawal may be a promising treatment strategy for KRAS^G12C^ mutant cancers. Given that DDR activation was noted after sotorasib withdrawal, the synergistic effects of inhibitors targeting DDR or DNA repair effectors during sotorasib withdrawal should be assessed in future studies.

## Conclusions

In conclusion, we identified the phenomenon of drug addiction in the sotorasib-treated cancer and identified the underlying mechanisms. The results provided the rationale of the drug holiday approach for sotorasib. The combination of sotorasib with a type I BRAF inhibitor may enhance sotorasib's drug addiction effect and may provide clinical benefits for patients with cancer.

## Supplementary Information

**Additional file 1: Table S1.** Antibodies used for Western blotting. **Table S2.** Antibodies used for immunofluorescence staining. **Table S3.** STR profiling of MIA PaCa-2 and MIA-SR. **Table S4.** STR profiling of NCI-H23 and H23-SR. **Table S5.** STR profiling of LU65 and 65-SR. **Table S6.** Non-silent genetic variants present in sotorasib-resistant cells but not present in parental cells identified by whole exome sequencing. **Figure S1**. Relative growth rates of MIA-PaCa-2, NCI-H23, and LU65 cells and their sotorasib-resistantsublines MIA-SR, H23-SR, and 65-SR cultured in the different concentrations of sotorasib for 3 days. Data are presented in terms of the fold-change values relative to the growth rates of cells cultured without sotorasib. ****P < 0.0001. **Figure S2**. Relative growth rates of the 65-SR cells cultured with or without 5 μM sotorasib. Data were normalized to the cell numbers noted after the 10-day-long treatment. **Figure S3**. A Quantification of the total ROS levels in the MIA-SR, H23-SR, and 65-SR cells cultured with or without 5 μM sotorasib for 3 days; the levels were measured through CellROX staining and flow cytometry. B The MIA-SR and H23-SR cells were cultured with or without 5 μM sotorasib in the presence of 2 mM NAC. The cells were counted on Day 11. Data are presented in terms of the mean ± standard deviation values of three cell culture replicates. ***P < 0.001, and ****P < 0.0001. **Figure S4**. Phase-contrast microscopy images of the MIA-SR and H23-SR cells cultured with or without 5 μM sotorasib for 8 days. **Figure S5.** Time-lapse microscopy images of the H23-SR cells cultured with or without 5 μM sotorasib for the indicated days. The yellow arrows denote mitotic cells. **Figure S6.** Effects of encorafenib on levels of p-ERK, ERK, p-RPA32, p-CHEK1, and p21 in the H23-SR cells levels with or without 5 μM sotorasib; protein levels were measured through Western blotting. **Figure S7.** Representative images of immunofluorescence staining and quantification of nucleoplasmic bridges and micronuclei in the H23-SR cells cultured with or without 5 μM sotorasib, 100 nM encorafenib, or both. The yellow arrows denote the locations of micronucleior nucleoplasmic bridges. *P < 0.05, ***P < 0.001, and ****P < 0.0001. **Figure S8.** Results of annexin V/PI flow cytometric assay on the H23-SR cells cultured with or without 5 μM sotorasib, 100 nM encorafenib, or both. **Figure S9**. Effects of sotorasib withdrawal on the levels of p-MEK1/2 and p-ERK in the 65-SR cells; protein levels were measured through Western blotting. **Figure S10**. Effects of encorafenib on tumor sizes and body weights of experimental mice. The 65-SR cells were subcutaneously implanted into the flanks of 6-week-old female NOD/SCID mice. Sotorasib was orally administered at 10 mg/kg/day to all mice one day before injection and throughout the experiment. After the mean tumor volume reached at least 100 mm^3^, the mice were treated with encorafenib or vesicle control treated for 14 days. Tumor sizes and body weights of the mice were measured at the indicated time point.

**Additional file 2: Movie 1.** Time-lapse microscopy images of the MIA-SR cells cultured with 5 μM sotorasib.

**Additional file 3: Movie 2.** Time-lapse microscopy images of the MIA-SR cells cultured without sotorasib for 8 days.

**Additional file 4: Movie 3.** Time-lapse microscopy images of the H23-SR cells cultured with 5 μM sotorasib.

**Additional file 5: Movie 4.** Time-lapse microscopy images of the H23-SR cells cultured without sotorasib for 13 days.

## Data Availability

All data generated during this study are included in this published article and its additional information files.

## Abbreviations

KRAS: Kirsten rat sarcoma viral oncogene homologue
NSCLC: Non-small-cell lung cancer
MAPK: Mitogen-activated protein kinase
PI3K: Phosphatidylinositol 3-kinase
NRAS: Neuroblastoma RAS viral oncogene homolog
BRAF: V-Raf murine sarcoma viral oncogene homolog B
EGFR: Epidermal growth factor receptor
FGFR2: Fibroblast growth factor receptor 2
MEK: Mitogen-activated protein kinase
DDR: DNA damage response
p21: P21^Waf1/Cip1^
FBS: Fetal bovine serum
STR: Short-tandem repeat
QVD: Q-VD-OPh
NAC: N-acetyl-l-cysteine
SR: Sotorasib-resistant
PBS: Phosphate-buffered saline
PI: Propidium iodide
BrdU: 5-Bromo-2′-deoxyuridine
BSA: Bovine serum albumin
γH2AX: Phospho-histone variant H2A.X (S139)
DAPI: 4′,6′-Diamidino-2-phenylindole
EdU: 5-Ethynyl-2′-deoxyuridine
ERK1/2: Extracellular-signal-regulated kinase 1/2
ROS: Reactive oxidative species
RPA: Replication protein A
ATR: Ataxia telangiectasia and Rad3-related protein
CHEK1: Checkpoint kinase 1
ATM: Ataxia-telangiectasia mutated kinase
DSB: Double strand break
BID: BH3 interacting-domain death agonist
AIF: Apoptosis-inducing factor
PARP-1: Poly (ADP-ribose) polymerase 1
CDC6: Cell division cycle 6
