# Supplementary material for: DNA replication stress and mitotic catastrophe mediate sotorasib addiction in KRASG12C-mutant cancer
Source: J Biomed Sci. 2023 Jun 29;30:50. doi: 10.1186/s12929-023-00940-4 (PMC10308767; doi:10.1186/s12929-023-00940-4)
Supplement: Supplementary file 1 — Additional file 1: Table S1. Antibodies used for Western blotting. Table S2. Antibodies used for immunofluorescence staining. Table S3. STR profiling of MIA PaCa-2 and MIA-SR. Table S4. STR profiling of NCI-H23 and H23-SR. Table S5. STR profiling of LU65 and 65-SR. Table S6. Non-silent genetic variants present in sotorasib-resistant cells but not present in parental cells identified by whole exome sequencing. Figure S1. Relative growth rates of MIA-PaCa-2, NCI-H23, and LU65 cells and their sotorasib-resistantsublines MIA-SR, H23-SR, and 65-SR cultured in the different concentrations of sotorasib for 3 days. Data are presented in terms of the fold-change values relative to the growth rates of cells cultured without sotorasib. ****P < 0.0001. Figure S2. Relative growth rates of the 65-SR cells cultured with or without 5 μM sotorasib. Data were normalized to the cell numbers noted after the 10-day-long treatment. Figure S3. A Quantification of the total ROS levels in the MIA-SR, H23-SR, and 65-SR cells cultured with or without 5 μM sotorasib for 3 days; the levels were measured through CellROX staining and flow cytometry. B The MIA-SR and H23-SR cells were cultured with or without 5 μM sotorasib in the presence of 2 mM NAC. The cells were counted on Day 11. Data are presented in terms of the mean ± standard deviation values of three cell culture replicates. ***P < 0.001, and ****P < 0.0001. Figure S4. Phase-contrast microscopy images of the MIA-SR and H23-SR cells cultured with or without 5 μM sotorasib for 8 days. Figure S5. Time-lapse microscopy images of the H23-SR cells cultured with or without 5 μM sotorasib for the indicated days. The yellow arrows denote mitotic cells. Figure S6. Effects of encorafenib on levels of p-ERK, ERK, p-RPA32, p-CHEK1, and p21 in the H23-SR cells levels with or without 5 μM sotorasib; protein levels were measured through Western blotting. Figure S7. Representative images of immunofluorescence staining and quantification of nucleoplasmic [file 12929_2023_940_MOESM1_ESM.docx]

**Additional file 1: information for**

DNA Replication Stress and Mitotic Catastrophe Mediate Sotorasib Addiction in KRAS^G12C^-Mutant Cancer

Li-Wen Chiou,^1^ Chien-Hui Chan,^1^ Yu-Ling Jhuang,^1^ Ching-Yao Yang,^2,3^ and Yung-Ming Jeng^1,4^

Corresponds to Ching-Yao Yang (cyang@ntuh.gov.tw) or Yung-Ming Jeng (mrna0912@gamil.com)

This document includes:

Additional file 1: Table S1 to S6

Additional file 1: Fig. S1 to S10

| Additional file 1: Table S1. Antibodies used for Western blotting | | | |
| --- | --- | --- | --- |
| Antibody | Dilution | Catalog No. | Brand |
| p-Erk1/2 (T202/Y204) | 1:1000 | 9101 | Cell Signaling Technology |
| Erk1/2 | 1:1000 | 9102 | Cell Signaling Technology |
| p-MEK1/2 (Ser217/221) | 1:1000 | 9154 | Cell Signaling Technology |
| MEK1/2 | 1:1000 | 9122 | Cell Signaling Technology |
| Cleaved caspase 3 | 1:2000 | 9661 | Cell Signaling Technology |
| p-RPA32 (S8) | 1:1000 | 54762 | Cell Signaling Technology |
| p-ATM (S1981) | 1:1000 | 4526 | Cell Signaling Technology |
| p-ATR (S428) | 1:1000 | 2853 | Cell Signaling Technology |
| p-CHEK1 (S345) | 1:1000 | 2348 | Cell Signaling Technology |
| p21 Waf1/Cip1 | 1:1000 | 2947 | Cell Signaling Technology |
| GAPDH | 1:6000 | GTX100118 | Genetex |

| Additional file 1: Table S2. Antibodies used for immunofluorescence staining. | | | |  |
| --- | --- | --- | --- | --- |
| Antibody | Dilution | Catalog No. | Brand | |
| p-Histone H2AX (S139)  (also known as γH2AX) | 1:2000 | 9718 | Cell Signaling Technology | |
| β-tubulin | 1:1000 | 86298 | Cell Signaling Technology | |
| γ-Tubulin | 1:1000 | ab11317 | Cell Signaling Technology | |
| p-Histone H3 (S10) | 1:1000 | 9706 | Cell Signaling Technology | |
| Alexa Fluor® 594  goat anti-rabbit | 1:500 | A-11012 | Thermo Fisher Scientific | |
| Alexa Fluor® 488  rabbit anti-mouse | 1:500 | A-11059 | Thermo Fisher Scientific | |
| Alexa Fluor® 647  p-Histone H2AX (S139) | 1:100 | 613407 | BioLegend | |
| Alexa Fluor® 488  p-Histone H3 (S10) | 1:100 | 650803 | BioLegend | |
| Alexa Fluor® 555  cleaved caspase 3 (D175) | 1:50 | 9604 | Cell Signaling Technology | |

| Additional file 1: Table S3. STR profiling of MIA PaCa-2 and MIA-SR cells. | | | |  |  |
| --- | --- | --- | --- | --- | --- |
| STR Loci | Sample Name | | Concordance (Yes/No) | |  |
|  | MIA PaCa-2 | MIA-SR |  |  |  |
| D8S1179 | 16,16 | 16,16 | Yes | |  |
| D21S11 | 29,31,2 | 29,31,2 | Yes | |  |
| D7S820 | 12,12 | 12,13 | No | |  |
| CSF1PO | 10,10 | 10,10 | Yes | |  |
| D3S1358 | 16,16 | 16,16 | Yes | |  |
| TH01 | 9,10 | 9,10 | Yes | |  |
| D13S317 | 12,13 | 12,13 | Yes | |  |
| D16S539 | 10,13 | 10,13 | Yes | |  |
| D2S1338 | 25,25 | 25,25 | Yes | |  |
| D19S433 | 15,15 | 15,15 | Yes | |  |
| vWA | 15,15 | 15,15 | Yes | | |
| TPOX | 9,9 | 9,9 | Yes | | |
| D18S51 | 12,12 | 12,12 | Yes | | |
| Amelogenin | X,X | X,X | Yes | | |
| D5S818 | 12,13 | 12,13 | Yes | | |
| FGA | 22,22 | 22,22 | Yes | | |

- Conclusion: Based on 16 STR loci comparison, 15 STR loci were concordant and 1 STR locus was different. Therefore, the identity between MIA PaCa-2 and MIA-SR was 96.9%.

| Additional file 1: Table S4. STR profiling of NCI-H23 and H23-SR cells. | | | |  |  |
| --- | --- | --- | --- | --- | --- |
| STR Loci | Sample Name | | Concordance (Yes/No) | |  |
|  | NCI-H23 | H23-SR |  |  |  |
| D8S1179 | 15,15 | 15,15 | Yes | |  |
| D21S11 | 30,30 | 30,30 | Yes | |  |
| D7S820 | 9,10 | 9,10 | Yes | |  |
| CSF1PO | 10,10 | 10,10 | Yes | |  |
| D3S1358 | 15,15 | 15,15 | Yes | |  |
| TH01 | 6,6 | 6,6 | Yes | |  |
| D13S317 | 12,12 | 12,12 | Yes | |  |
| D16S539 | 11,11 | 11,11 | Yes | |  |
| D2S1338 | 18,23 | 18,23 | Yes | |  |
| D19S433 | 12,14 | 12,14 | Yes | |  |
| vWA | 16,17 | 16,17 | Yes | | |
| TPOX | 8,9 | 8,9 | Yes | | |
| D18S51 | 14,14 | 14,14 | Yes | | |
| Amelogenin | X,X | X,X | Yes | | |
| D5S818 | 12,13 | 12,13 | Yes | | |
| FGA | 24,24 | 24,24 | Yes | | |

- Conclusion: Based on 16 STR loci comparison, all 16 STR loci were concordant. Therefore, the identity between NCI-H23 and H23-SR was 100%.

| Additional file 1: Table S5. STR profiling of LU65 and 65-SR cells. | | | |  |  |
| --- | --- | --- | --- | --- | --- |
| STR Loci | Sample Name | | Concordance (Yes/No) | |  |
|  | LU65 | 65-SR |  |  |  |
| D8S1179 | 14,14 | 14,14 | Yes | |  |
| D21S11 | 31.2,31.2 | 31.2,31.2 | Yes | |  |
| D7S820 | 11,12 | 11,12 | Yes | |  |
| CSF1PO | 10,13 | 10,13 | Yes | |  |
| D3S1358 | 15,16 | 15,16 | Yes | |  |
| TH01 | 6,6 | 6,6 | Yes | |  |
| D13S317 | 10,10 | 10,10 | Yes | |  |
| D16S539 | 9,11 | 9,11 | Yes | |  |
| D2S1338 | 17,26 | 17,26 | Yes | |  |
| D19S433 | 11,15.2 | 11,15.2 | Yes | |  |
| vWA | 18,18 | 18,18 | Yes | | |
| TPOX | 8,11 | 8,11 | Yes | | |
| D18S51 | 18,18 | 18,18 | Yes | | |
| Amelogenin | X,X | X,X | Yes | | |
| D5S818 | 12,12 | 12,12 | Yes | | |
| FGA | 26,26 | 26,26 | Yes | | |

- Conclusion: Based on 16 STR loci comparison, all 16 STR loci were concordant. Therefore, the identity between NCI-H23 and H23-SR was 100%.

Additional file 1: **Table S6.** Non-silent genetic variants present in sotorasib-resistant cells but not present in parental cells identified by whole exome sequencing.

| Cell | Chromo  some | Region | Type | Reference | Allele | Frequency | gene | Amino acid change |
| --- | --- | --- | --- | --- | --- | --- | --- | --- |
| MIA PaCa-2 | 4 | 25018092 | SNV | C | A | 61.40351 | LGI2 | p.Met184Ile |
| MIA PaCa-2 | 4 | 46097276 | SNV | G | A | 44.89796 | GABRG1 | p.His60Tyr |
| MIA PaCa-2 | 7 | 100373690 | SNV | T | C | 36.78161 | PILRA |  |
| MIA PaCa-2 | 7 | 149765079 | SNV | C | T | 38.46154 | ZNF467 | p.Val689Ile |
| MIA PaCa-2 | 7 | 150372307..  150372318 | Deletion | CCGCCAG  GGGCC | - | 40 | REPIN1 | p.Gly358_Pro361del |
| MIA PaCa-2 | 7 | 150720726 | SNV | G | A | 35.29412 | GIMAP1 | p.Arg241Gln |
| MIA PaCa-2 | 8 | 132873145 | SNV | G | A | 39.24051 | TG | p.Glu188Lys |
| MIA PaCa-2 | 13 | 19646444 | SNV | G | T | 39.13043 | MPHOSPH8 | p.Arg124Ile |
| MIA PaCa-2 | 17 | 8263564..8263581 | Deletion | CCTCTCACCA  GGCAATGT | - | 37.28814 | PFAS | p.Gly523fs |
| MIA PaCa-2 | X | 148662130 | SNV | C | A | 39.47368 | AFF2 | p.Pro135Thr |
| LU65 | 1 | 155322797 | SNV | T | C | 35.71429 | RUSC1 | p.Trp342Arg |
| LU65 | 1 | 157697665 | SNV | C | T | 38.02817 | FCRL3 | p.Val185Ile |
| LU65 | 2 | 27653709 | SNV | G | T | 52 | SUPT7L | p.His339Asn |
| LU65 | 2 | 39666311 | SNV | G | T | 72.72727 | TMEM178A | p.Gly113Cys |
| LU65 | 2 | 43224601..43224603 | Deletion | CTG | - | 40 | ZFP36L2 | p.Gln401del |
| LU65 | 3 | 121622497 | SNV | C | A | 52.77778 | FBXO40 | p.Tyr356* |
| LU65 | 3 | 191275324 | SNV | G | T | 43.39623 | UTS2B | p.Leu88Ile |
| LU65 | 4 | 56931099 | SNV | A | G | 43.93939 | REST | p.Ile747Met |
| LU65 | 9 | 77260121 | SNV | T | C | 49.01961 | VPS13A | p.Leu775Se |
| LU65 | 9 | 114168642 | SNV | A | G | 41.48936 | COL27A1 | p.Lys363Glu |
| LU65 | 11 | 1095999 | SNV | A | C | 35.29412 | MUC2 | p.Gln1919Pro |
| LU65 | 11 | 8038911 | SNV | C | T | 53.62319 | TUB | p.Ser13Phe |
| LU65 | 11 | 9424988 | SNV | G | T | 55.55556 | IPO7 | p.Glu406* |
| LU65 | 11 | 102955493 | SNV | G | A | 59.01639 | MMP13 | p.Arg41Cys |
| LU65 | 15 | 89776890..  89776913 | Deletion | AGGGGCAGG  GGCAAGGGC  AGGGGC | - | 36.36364 | MESP2 | p.Gln198_Gly205del |
| LU65 | 19 | 501743^501744 | Insertion | - | CTCCCGACAC  CACCTCCCCG  GAGT | 47.2 | MADCAM1 | p.Ser253_  Gln254insPro  GluSerProAsp  ThrThrSer |
| LU65 | 19 | 7870909..7870977 | Deletion | GGGGTGTG  GTCAGGGGAGCA  GAAGCTGTCTGCA  GAGGAGGCGGG  GCTAGAGAAGGT  AGGTTCTCCAGAG | - | 46.15385 | PRR36 | p.Pro756_Pro778del |
| LU65 | 19 | 46756585 | SNV | C | T | 37.43842 | FKRP | p.Arg379Trp |
| LU65 | X | 139814960 | SNV | C | G | 47.61905 | ATP11C | p.Arg115Thr |
| LU65 | X | 141242395..141242396 | MNV | GG | CA | 100 | SPANXC | p.Ala21Val |
|  |  |  |  |  |  |  |  |  |
| H23 | 2 | 27101386..27101436 | Deletion | GCCTCTGCCTGGCCCCCAGCTTCCCCTCTGGGCCCGGGGGCATCTCCTTCA | - | 40.90909 | CGREF1 | p.Glu266_Ala282del |
| H23 | 2 | 232408794 | SNV | T | G | 100 | ALPG | p.Leu316Arg |
| H23 | 4 | 1394562^1394563 | Insertion | - | TGCCCATGTGGAGTGCCCGCCTGCTCACACA | 91.86047 | CRIPAK | p.Cys27fs |
| H23 | 6 | 37022289 | SNV | G | T | 40.17857 | FGD2 | p.Lys459Asn |
| H23 | 8 | 8797426 | SNV | C | T | 52.26337 | MFHAS1 | p.Glu1022Lys |
| H23 | 9 | 35106550..35106551 | MNV | CA | AG | 63.85542 | FAM214B | p.Leu349Pro |
| H23 | 9 | 76296854 | SNV | A | G | 39.8374 | PCSK5 | p.Lys725Arg |
| H23 | 10 | 27403299 | SNV | A | C | 40.8377 | PTCHD3 | p.Leu424Val |
| H23 | 11 | 61766157 | Deletion | A | - | 44 | MYRF | p.Lys112fs |
| H23 | 13 | 77098918 | SNV | C | T | 56.70103 | MYCBP2 | p.Gly2865Arg |
| H23 | 14 | 89953985 | SNV | T | C | 45.45455 | EFCAB11 | p.Asp7Gly |
| H23 | 14 | 90184689 | SNV | G | A | 57.14286 | KCNK13 | p.Gly305Arg |
| H23 | 14 | 90676655 | SNV | G | C | 58.41584 | TTC7B | :p.Asn340Lys |
| H23 | 14 | 91610481 | SNV | G | A | 46.15385 | CATSPERB | p.Pro866Leu |
| H23 | 14 | 92071010^92071011 | Insertion | - | CTGCTGCTGCTGCTGCTGCTGCTGCTGCTGCTGCTGCTG | 76.92308 | ATXN3 | p.Gln305[14] |
| H23 | 14 | 92456487 | SNV | T | A | 53.71901 | SLC24A4 | p.Asn406Lys |
| H23 | 14 | 93612859 | SNV | G | A | 52.88462 | UNC79 | p.Asp1340Asn |
| H23 | 14 | 94001151 | SNV | C | T | 48.71795 | CCDC197 | p.Thr65Met |
| H23 | 14 | 94003235 | SNV | C | T | 52.52525 | CCDC197 | p.Arg127Trp |
| H23 | 14 | 94381078 | SNV | A | G | 36.36364 | SERPINA1 | p.Val237Ala |
| H23 | 14 | 94446367 | SNV | T | C | 37.7551 | SERPINA11 | p.Gln294Arg |
| H23 | 14 | 94496377 | SNV | G | A | 49.48454 | SERPINA12 | p.Arg301Cys |
| H23 | 14 | 95436971..95436973 | Deletion | AGA | - | 39.74359 | SYNE3 | p.Leu796del |
| H23 | 14 | 95439984 | SNV | G | A | 50 | SYNE3 | p.Thr668Met |
| H23 | 14 | 95690850 | SNV | G | A | 52.63158 | TCL1B | p.Gly93Arg |
| H23 | 14 | 96264632 | SNV | G | A | 46.57534 | BDKRB1 | p.Arg317Gln |
| H23 | 14 | 102229356 | SNV | T | C | 36.76471 | MOK | p.Gln164Arg |
| H23 | 14 | 102335758 | SNV | A | T | 36.73469 | ZNF839 | p.Met457Leu |
| H23 | 14 | 103100437 | SNV | C | T | 50.61728 | EXOC3L4 | p.Thr131Met |
| H23 | 14 | 103133510 | SNV | C | T | 54.05405 | TNFAIP2 | p.Thr565Ile |
| H23 | 14 | 103475055 | SNV | A | G | 35.48387 | MARK3 | p.Ser429Gly |
| H23 | 14 | 103563041 | SNV | C | G | 46.875 | COA8 | p.Pro14Ala |
| H23 | 14 | 104177521..104177522 | MNV | GA | AG | 44.44444 | KIF26A | p.Arg1578Gln |
| H23 | 14 | 104588706 | SNV | C | T | 66.66667 | C14orf180 | p.Arg136Trp |
| H23 | 14 | 104714640 | SNV | G | A | 47.61905 | INF2 | p.Gly1160Ser |
| H23 | 14 | 104946731 | SNV | G | C | 37.5 | AHNAK2 | p.Pro2807Arg |
| H23 | 14 | 104949204 | SNV | T | C | 40.90909 | AHNAK2 | p.Lys1983Glu |
| H23 | 14 | 104949883 | SNV | T | A | 39.02439 | AHNAK2 | p.Glu1756Asp |
| H23 | 14 | 104950863 | SNV | G | A | 61.53846 | AHNAK2 | p.Pro1430Ser |
| H23 | 14 | 105469487 | SNV | G | A | 52.17391 | MTA1 | p.Ala672Thr |
| H23 | 15 | 68205259 | SNV | G | A | 63.08725 | CALML4 | p.Arg40* |
| H23 | 15 | 68335711 | SNV | T | G | 64 | ITGA11 | p.Met471Leu |
| H23 | 15 | 69047494 | SNV | G | A | 47.27273 | NOX5 | p.Gly557Ser |
| H23 | 15 | 73927241 | SNV | G | A | 72.72727 | LOXL1 | p.Gly153Asp |
| H23 | 15 | 73994894 | SNV | A | C | 70.27027 | PML | p.Thr28Pro |
| H23 | 15 | 74722351..74722352 | MNV | AT | CA | 59.48276 | CYP1A1 | p.Asn249Met |
| H23 | 15 | 74751251 | SNV | C | A | 65.5814 | CYP1A2 | p.Ser298Arg |
| H23 | 15 | 74802439 | SNV | G | A | 58.18182 | CSK | p.Ala427Thr |
| H23 | 15 | 74819189 | SNV | G | A | 68.53933 | LMAN1L | p.Gly212Asp |
| H23 | 15 | 75207520 | SNV | G | A | 67.41573 | C15orf39 | p.Gly491Asp |
| H23 | 15 | 75207654 | SNV | T | G | 64.28571 | C15orf39 | p.Ser536Ala |
| H23 | 15 | 76203891 | SNV | G | A | 62.85714 | TMEM266 | p.Arg391His |
| H23 | 15 | 76203999 | SNV | C | T | 65.625 | TMEM266 | p.Pro427Leu |
| H23 | 15 | 76204086 | SNV | A | G | 66.25 | TMEM266 | p.Lys456Arg |
| H23 | 15 | 77037069 | SNV | G | A | 67.08861 | PSTPIP1 | p.Ala373Thr |
| H23 | 15 | 78487762 | SNV | T | C | 67.08861 | IREB2 | p.Ile523Thr |
| H23 | 15 | 79971003 | SNV | A | C | 65.18987 | BCL2A1 | p.Asn39Lys |
| H23 | 15 | 80591684 | SNV | G | A | 70.40816 | ARNT2 | p.Gly679Ser |
| H23 | 15 | 82220088 | SNV | C | G | 58.16327 | EFL1 | p.Glu478Asp |
| H23 | 15 | 84783285 | SNV | G | T | 68.78613 | ZNF592 | p.Glu204* |
| H23 | 15 | 84798628 | SNV | G | A | 56.55738 | ZNF592 | p.Ser926Asn |
| H23 | 15 | 85244728 | SNV | G | T | 57.14286 | GOLGA6L3 | p.Gln246His |
| H23 | 15 | 85247091 | SNV | C | T | 38.18182 | GOLGA6L3 | p.Ala505Val |
| H23 | 15 | 85579423 | SNV | T | C | 60.19417 | AKAP13 | p.Met452Thr |
| H23 | 15 | 85579548 | SNV | T | C | 58.33333 | AKAP13 | p.Trp494Arg |
| H23 | 15 | 85579788 | SNV | C | T | 57.04225 | AKAP13 | p.Arg574Cys |
| H23 | 15 | 85580602 | SNV | T | C | 62.83186 | AKAP13 | p.Val845Ala |
| H23 | 15 | 85580757 | SNV | G | A | 70.68966 | AKAP13 | p.Val897Met |
| H23 | 15 | 85581252 | SNV | C | G | 61.03896 | AKAP13 | :p.Pro1062Ala |
| H23 | 15 | 85581324 | SNV | G | A | 60.57692 | AKAP13 | p.Asp1086Asn |
| H23 | 15 | 85581715 | SNV | T | C | 64.84375 | AKAP13 | p.Met1216Thr |
| H23 | 15 | 85744636 | SNV | G | A | 57.57576 | AKAP13 | p.Ala2805Thr |
| H23 | 15 | 88626627 | SNV | A | G | 66.66667 | AEN | p.Asn140Asp |
| H23 | 15 | 88838898 | SNV | C | A | 69.54023 | ACAN | p.Asp102Glu |
| H23 | 15 | 88849708 | SNV | G | A | 61.76471 | ACAN | p.Arg668Gln |
| H23 | 15 | 88857449 | SNV | A | G | 63.1068 | ACAN | p.Thr1622Ala |
| H23 | 15 | 88858997 | SNV | G | A | 60 | ACAN | p.Glu2138Lys |
| H23 | 15 | 89702023 | SNV | G | A | 72.95082 | WDR93 | p.Val93Ile |
| H23 | 15 | 89738221 | SNV | G | A | 56.45161 | WDR93 | p.Val430Ile |
| H23 | 15 | 89776769 | SNV | G | A | 68.11594 | MESP2 | p.Val138Met |
| H23 | 15 | 90067303 | SNV | A | G | 70.48193 | ZNF710 | p.Met56Val |
| H23 | 15 | 90220987 | SNV | G | A | 60.11236 | SEMA4B | p.Met163Ile |
| H23 | 15 | 90228555 | SNV | G | A | 57.14286 | SEMA4B | p.Ser809Asn |
| H23 | 15 | 90241630 | SNV | G | A | 77.68595 | GDPGP1 | p.Gly241Glu |
| H23 | 15 | 90910945 | SNV | C | T | 63.88889 | MAN2A2 | p.Ala620Val |
| H23 | 15 | 90916245 | SNV | G | A | 75.5102 | MAN2A2 | p.Val995Met |
| H23 | 15 | 90947796..90947797 | MNV | GG | TT | 64.28571 | UNC45A | p.Gly356Leu |
| H23 | 15 | 90953012 | SNV | C | T | 73.78641 | UNC45A | p.Thr651Met |
| H23 | 15 | 93045455 | SNV | T | A | 60.40268 | RGMA | p.Glu299Val |
| H23 | 15 | 99130060 | SNV | C | T | 64.97175 | SYNM | p.Pro567Leu |
| H23 | 15 | 99130272 | SNV | G | A | 71.18644 | SYNM | p.Val638Ile |
| H23 | 15 | 99729475 | SNV | G | C | 63.54167 | LYSMD4 | p.Ala181Gly |
| H23 | 15 | 101331974 | SNV | G | A | 70.86614 | PCSK6 | p.Thr639Ile |
| H23 | 15 | 101724101 | SNV | G | C | 64.28571 | TARSL2 | p.Ala96Gly |
| H23 | 22 | 39686155 | SNV | C | A | 92.85714 | CACNA1I | p.Pro1524Gln |


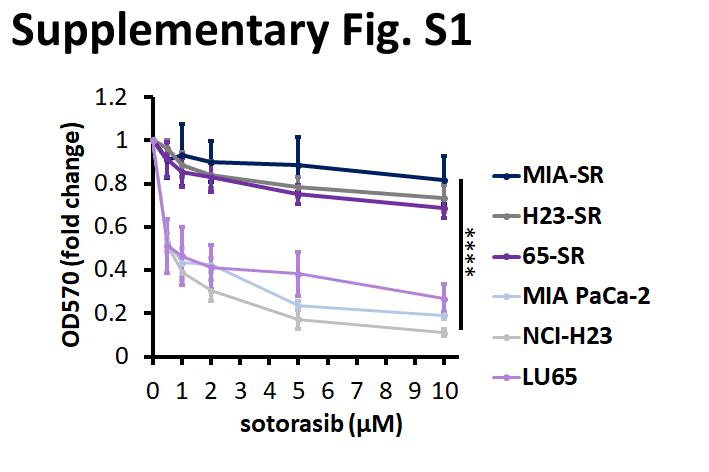


Additional file 1: **Fig. S1**. Relative growth rates of MIA-PaCa-2, NCI-H23, and LU65 cells and their sotorasib-resistant (SR) sublines MIA-SR, H23-SR, and 65-SR cultured in the different concentrations of sotorasib for 3 days. Data are presented in terms of the fold-change values relative to the growth rates of cells cultured without sotorasib. ****: *P* < 0.0001.


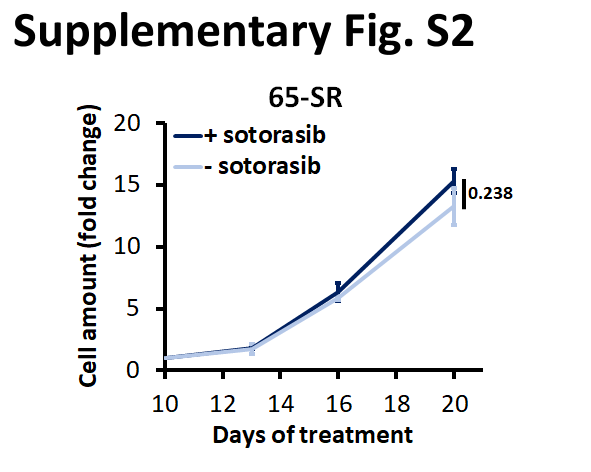


Additional file 1: **Fig. S2**. Relative growth rates of 65-SR cells cultured with or without 5 μM sotorasib. Data were normalized to the cell numbers noted after the 10-day-long treatment.


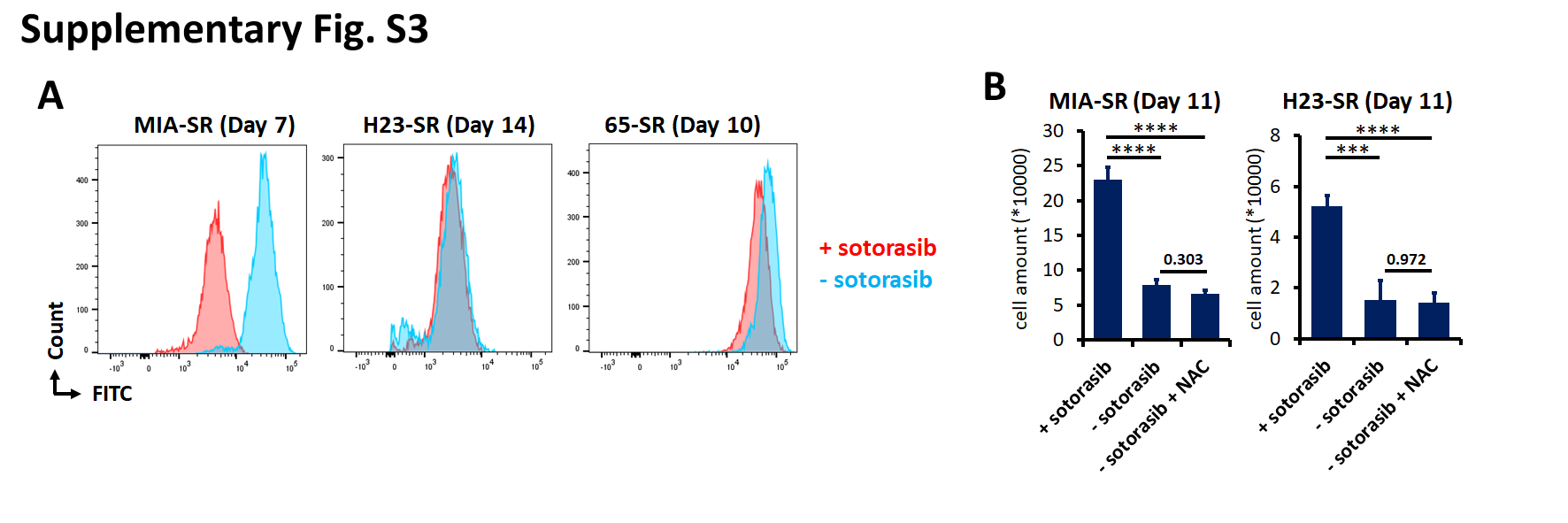


Additional file 1: **Fig. S3**. **A** Quantification of the total ROS levels in the MIA-SR, H23-SR, and 65-SR cells cultured with or without 5 μM sotorasib for 3 days; the levels were measured through CellROX staining and flow cytometry. **B** MIA-SR and H23-SR cells were cultured with or without 5 μM sotorasib in the presence of 2 mM N-acetyl-l-cysteine (NAC). The cells were counted on Day 11. Data are presented in terms of the mean ± standard deviation values of three cell culture replicates. ***: *P* < 0.001, and ****: *P* < 0.0001.


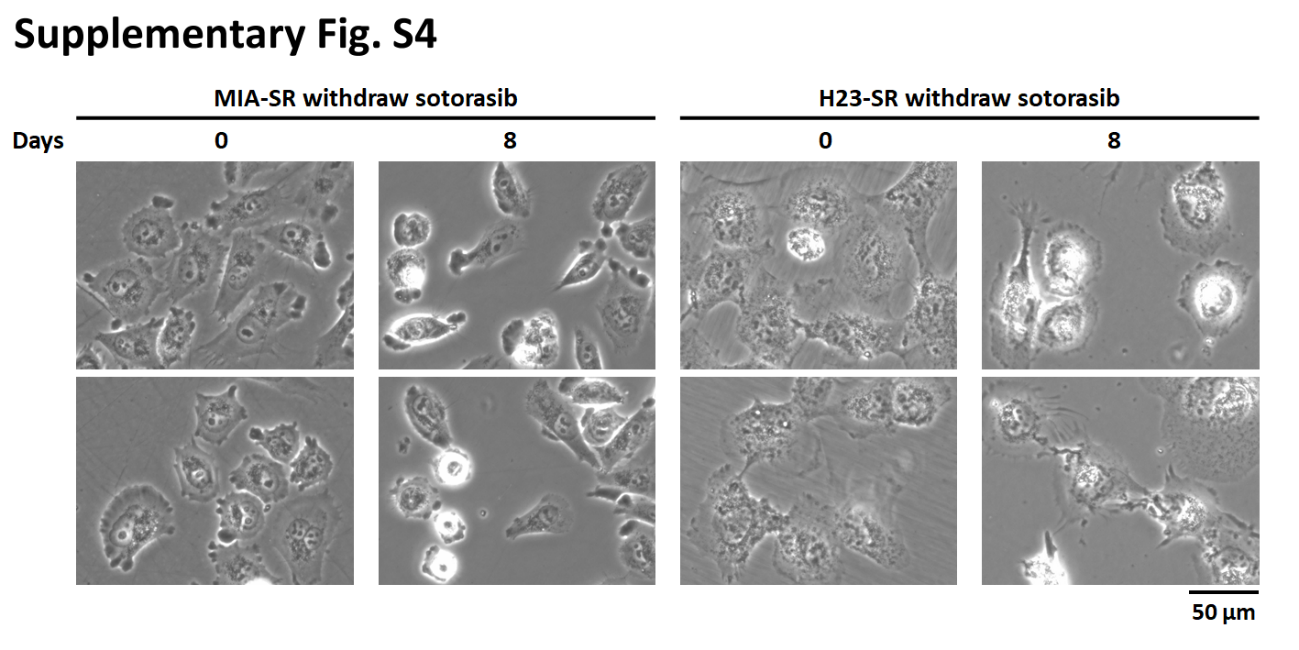


Additional file 1: **Fig. S4**. Phase-contrast microscopy images of MIA-SR and H23-SR cells cultured with or without 5 μM sotorasib for 8 days.


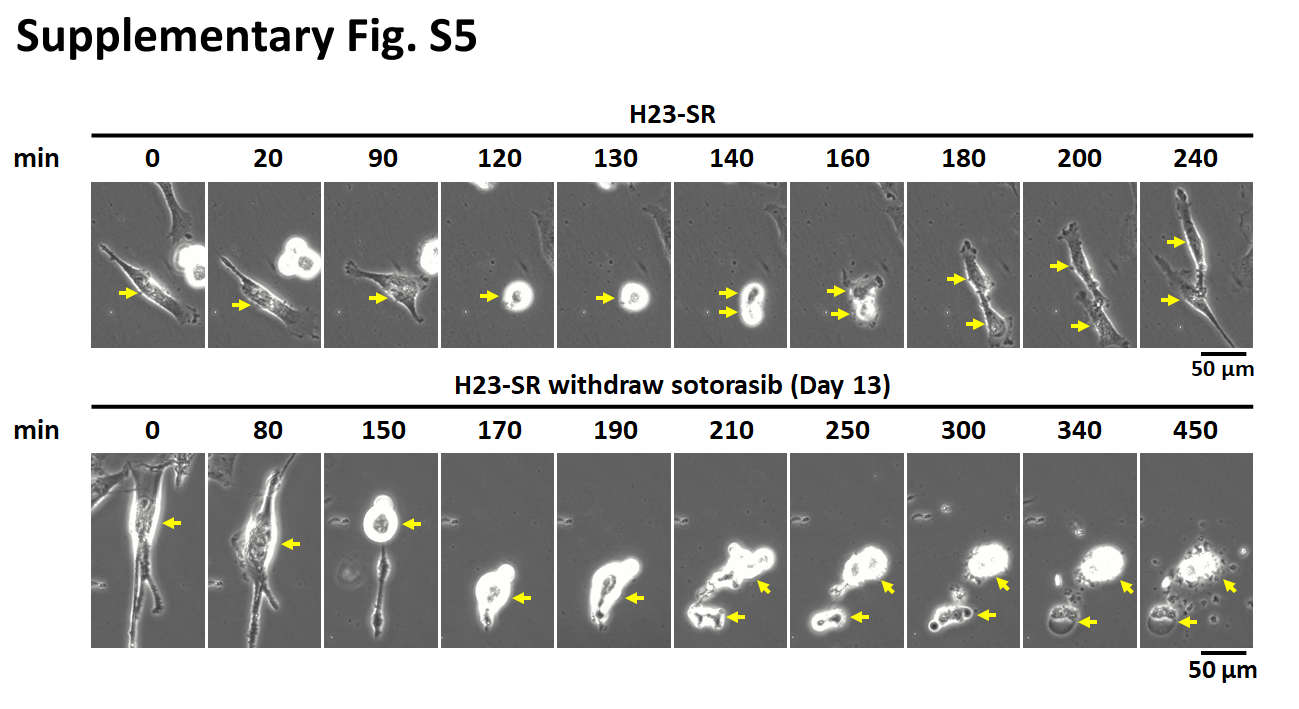


Additional file 1: **Fig. S5.** Time-lapse microscopy images of H23-SR cells cultured with or without 5 μM sotorasib for the indicated days. The yellow arrows denote mitotic cells.


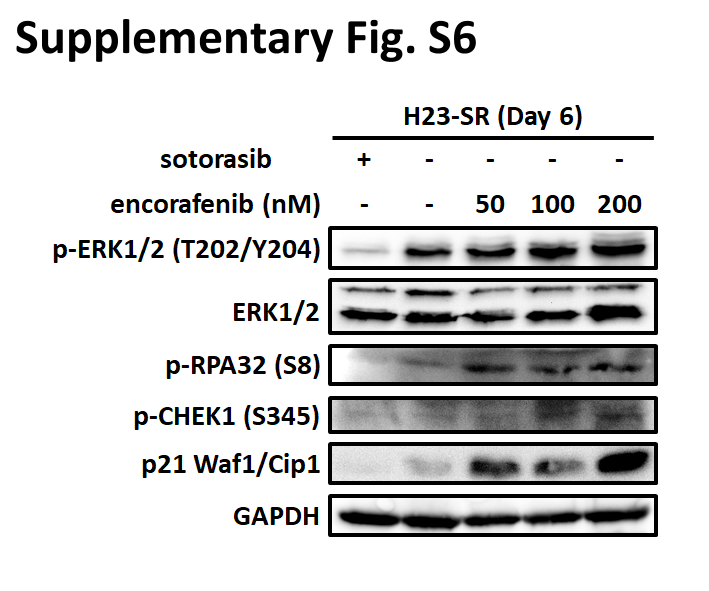


Additional file 1: **Fig. S6.** Effects of encorafenib on levels of p-ERK, ERK, p-RPA32, p-CHEK1, and p21 in H23-SR cells levels with or without 5 μM sotorasib; protein levels were measured through Western blotting.


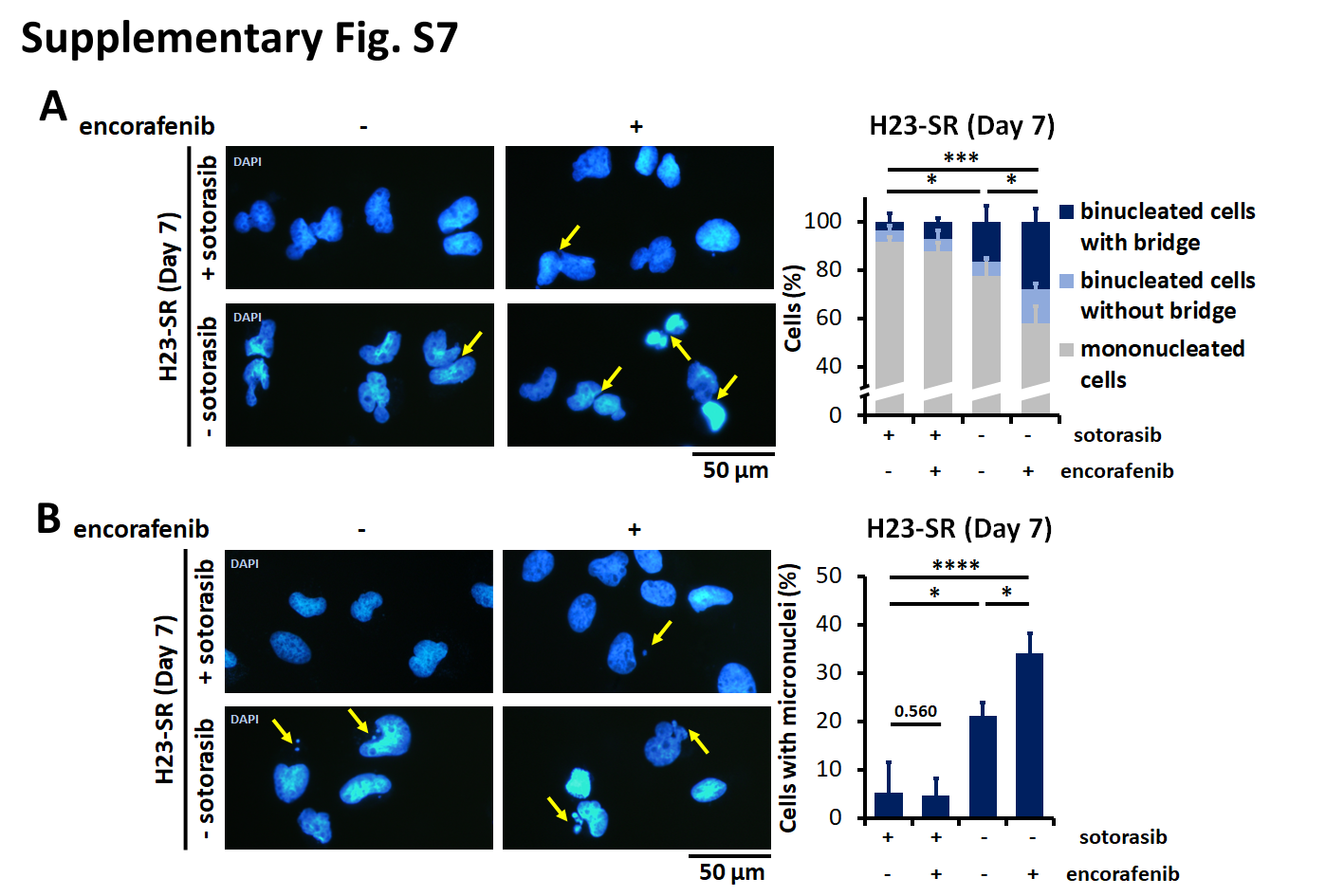


Additional file 1: **Fig. S7.** Representative images of immunofluorescence staining and quantification of nucleoplasmic bridges (**A**) and micronuclei (**B**) in H23-SR cells cultured with or without 5 μM sotorasib, 100 nM encorafenib, or both. The yellow arrows denote the locations of micronuclei (**A**) or nucleoplasmic bridges (**B**). *: *P* < 0.05, ***: *P* < 0.001, and ****: *P* < 0.0001.


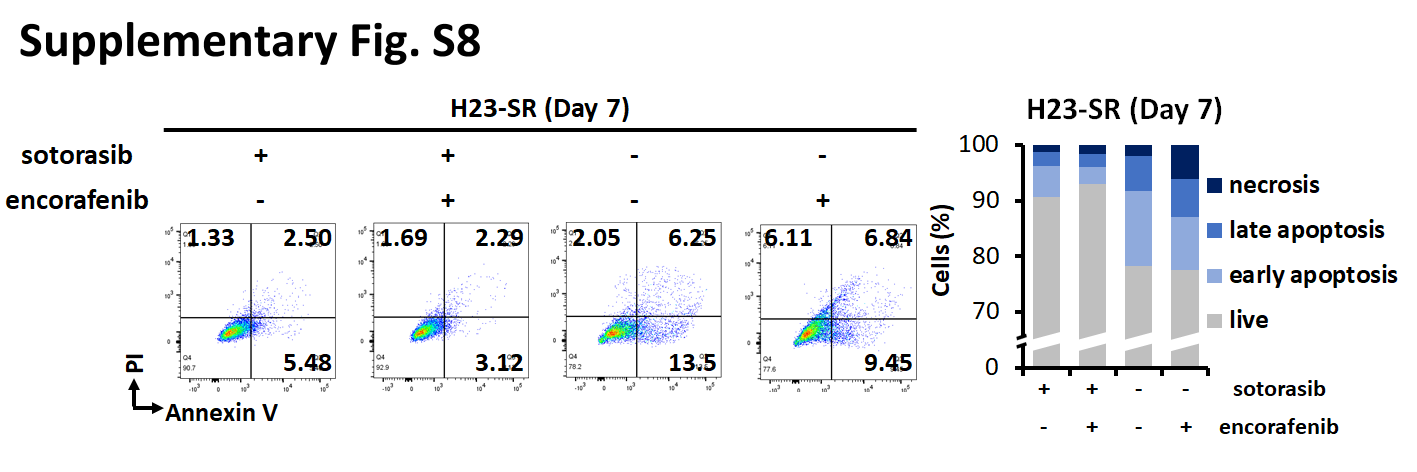


Additional file 1: **Fig. S8.** Results of annexin V/PI flow cytometric assay on H23-SR cells cultured with or without 5 μM sotorasib, 100 nM encorafenib, or both.


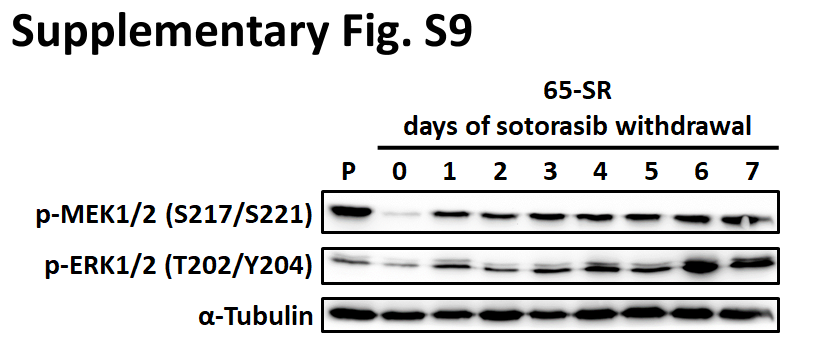


Additional file 1: **Fig. S9**. Effects of sotorasib withdrawal on the levels of p-MEK1/2 and p-ERK in 65-SR cells; protein levels were measured through Western blotting.


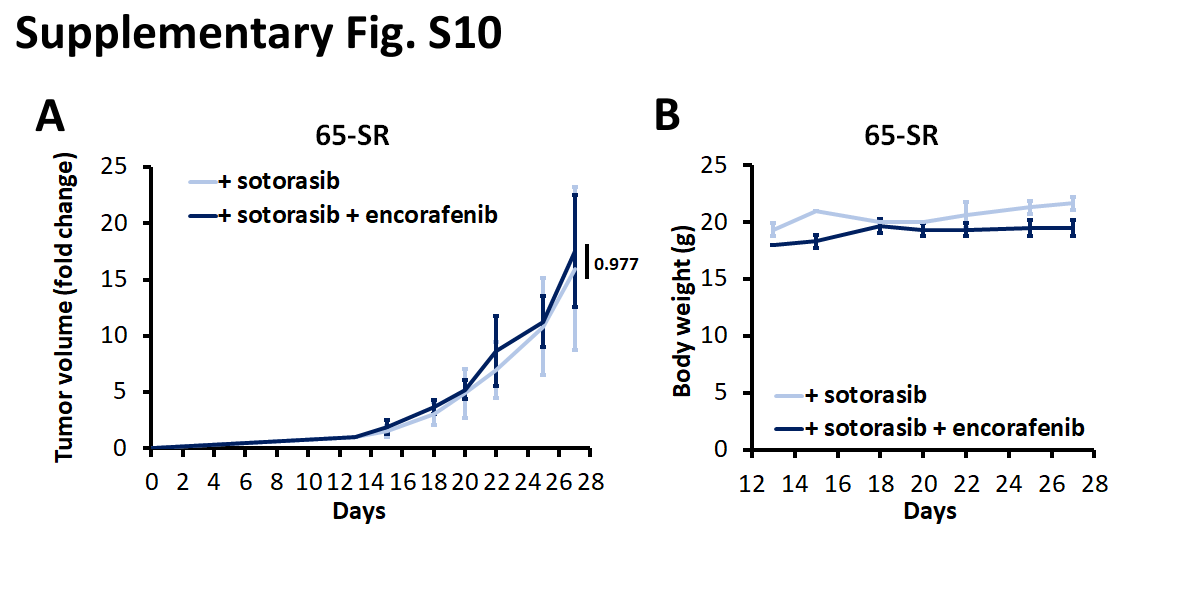


Additional file 1: **Fig. S10**. Effects of encorafenib on tumor sizes (**A**) and body weights (**B**) of experimental mice. 65-SR cells were subcutaneously implanted into the flanks of 6-week-old female NOD/SCID mice. Sotorasib was orally administered at 10 mg/kg/day to all mice one day before injection and throughout the experiment. After the mean tumor volume reached at least 100 mm^3^, the mice were treated with encorafenib (20 mg/kg daily) or vesicle control (n = 4-6 mice per group) treated for 14 days. Tumor sizes (**A**) and body weights (**B**) of the mice were measured at the indicated time point.
